# Impacts of rainforest fragmentation on the composition of ground-active vertebrate communities and their patterns of seed consumption

**DOI:** 10.1371/journal.pone.0202870

**Published:** 2018-09-12

**Authors:** Gary J. Palmer, Carla P. Catterall

**Affiliations:** 1 School of Environment, Griffith University, Nathan, Queensland, Australia; 2 Environmental Futures Research Institute, Griffith University, Nathan, Queensland, Australia; Auburn University, UNITED STATES

## Abstract

Post-dispersal seed consumption by rainforest vertebrates on the forest floor can substantially influence the community dynamics of rainforest trees. Studies of rainforest vertebrate seed predators at a community level, however, are lacking. Furthermore, there is very limited understanding of the effects of forest fragmentation on seed predators and their feeding behaviour. Here, we test whether communities of vertebrate seed predators, and their patterns of feeding on rainforest tree seeds, are altered when clearing creates forest fragments in an agricultural matrix. Using infra-red trail cameras deployed at stations with and without seeds of 20 local tree species, we identified four mammal and three bird species (from 18 recorded vertebrate taxa at mainly species level) as common post-dispersal seed predators in subtropical rainforest of eastern Australia. Statistical comparisons of species-specific frequencies between six sites in continuous forest and six in small rainforest fragments (4–21 ha) showed that habitat fragmentation substantially altered species composition of seed predator communities. Two species, both small rodents, had lower abundances in fragments than in continuous forest, while higher abundances were observed in fragments for a further four species: two small birds, a medium-sized marsupial and the small non-native rodent *Rattus rattus*. The abundance of one larger bird species did not change. Predatory interest in seeds was also significantly affected by habitat fragmentation and generally increased in each species’ habitat of greater abundance. Collectively, seed predators showed behaviours associated with potential or actual seed consumption on an average of 43% of camera days with seeds, with about 50% of seeds physically removed or damaged after five days’ exposure. Camera data have revealed community-level changes in seed predator abundance and feeding that are likely to cause altered patterns of plant recruitment following rainforest fragmentation, but these will be complex in nature.

## Introduction

Post-dispersal predation of seeds by rainforest vertebrates that forage on the forest floor can lead to high levels of seed mortality [1], [2]. In the neotropics, for example, rodents removed 61–77% of seeds of four rainforest tree species after 30 days exposure [3]. Declines in the abundance of common seed predator species can therefore lead to decreased rates of seed predation, in turn driving increased rates of plant recruitment. For example, experimental reduction in the density of a seed-eating rodent resulted in much lower rates of seed predation, and increased germination, of nine tree species in neotropical rainforest [4]. However, if such predator declines occur as part of turnover in community-level species composition, the potential changes in seed mortality and subsequent seedling recruitment may be reduced or offset by functional substitution through increased abundances of other seed predator species. For example, Galetti et al. [5] observed greater predation rates on palm seeds resulting from increases in either the abundance or feeding activity of several rodent species following local extinction of neotropical seed-eating peccaries. Research in this field, however, has typically focused on single species or taxa, especially rodents, which have been identified as functionally important seed predators in rainforests worldwide [2], [3], [6–12]. Knowledge of seed-seed predator interactions at community levels remains very limited.

In regions subjected to high levels of deforestation, much remaining forest now occurs as scattered fragments of habitat surrounded by a cleared matrix of pasture or agriculture, within which ground-active vertebrate communities can be substantially altered [13], [14]. Large-bodied vertebrates have often been at greater risk of reduced abundance or extinction in forest fragments than small-bodied species, at least in neotropical rainforests, [15–17]. However, the effects of fragmentation on the abundances of vertebrate species can be highly variable. For example, Fleury and Galetti [18] found that neotropical rainforest fragments smaller than 100 ha contained lower abundances of a small rodent (a squirrel < 1 kg) in association with degraded habitat quality. Similarly, Laurance [19] found that communities of small mammals (rodents and marsupials < 1 kg) in Australian tropical rainforest fragments were characterised by relatively lower abundances or absence of three common species that were forest-dependent. However, in the same study, four small matrix-tolerant species had greater abundances in fragments relative to larger areas of more continuous forest [19].

Changes in post-dispersal seed predation resulting from the modification of ground-active vertebrate communities in rainforest fragments are likely to lead to longer term changes in the floristic composition of vegetation, with subsequent impacts on the entire community. The concurrent impacts of fragmentation on both the abundance of vertebrate seed predator species and their contributions to rates of post-dispersal seed predation remain poorly understood, and have not previously been directly quantified. However, some insights have been provided by comparisons of differently-fragmented sites involving live-trapping studies of some seed-eating mammals. These studies have been combined with experimental exclosures and measurements of rates of removal or damage to seeds of a limited number of selected tree species [18], [20–24]. Such studies have revealed highly variable fragmentation responses among both seed-eating mammal species and seed predation rates. Granivorous birds have rarely been targeted for such investigations even though they may be ecologically significant post-dispersal seed predators within rainforest ecosystems [25]. Indeed, the effects of habitat fragmentation on whole communities of post-dispersal seed predators have not been directly studied in any region.

Automated motion-sensitive infra-red cameras (game or trail cameras) provide an opportunity to directly record both the presence and behaviours of vertebrates [26], enabling new types of investigation into both the species composition of, and predation rates by, seed-eating vertebrates. In the present study this camera technology, combined with experimental seed stations, was used to quantify interactions between ground-active vertebrate seed predator species and dispersed seeds, across multiple sites in Australian rainforest. In particular, we sought to examine community-wide effects of fragmentation on vertebrate seed predators and their seed predation behaviours. Specifically, we addressed the following questions. (1) What is the species composition of ground-active vertebrate seed predators? (2) To what extent do individual seed predator species contribute to rates of seed predation? (3) How does fragmentation alter both the vertebrate seed predator species composition and the predators’ patterns of feeding on seeds of a representative diversity of rainforest tree species?

## Materials and methods

### Study region and site network

Communities of ground-active vertebrate seed predators and their interactions with seeds were quantified in continuous and fragmented rainforest sites in the Big Scrub region of subtropical eastern Australia (28°35'-28°48'S, 153°10'-153°31'E; elevation 100–200 m asl). The region’s mean annual minimum and maximum temperatures are 13 °C and 26°C, and annual rainfall is 1343–2327 mm, being greatest during November-May. Prior to European settlement in the late 1800s, the region supported about 750 km^2^ of warm subtropical rainforest, mainly on deep basaltic soils, characterised by a high diversity of tall co-dominant tree species forming a closed but uneven canopy, with high representation of the Lauraceae, Malvaceae, Myrtaceae, Rutaceae and Sapindaceae families [27].

The subtropical rainforests of eastern Australia have been extensively cleared since European settlement, creating small fragments scattered across a highly modified landscape. In the Big Scrub, deforestation for agriculture had removed over 99% of the region’s original rainforest cover by the mid-1990s [27], [28]. The largest remnants (75–150 ha) are located the region’s northern end where they form part of a much larger uncleared forest habitat mosaic, interspersed with moist eucalypt forests and higher-elevation rainforests. Other rainforest remnants in the region are much smaller (< 20 ha) and are scattered through the agricultural landscape [28] which, at the time of the present study, was mostly livestock pasture with some areas of *Macadamia* sp. tree plantation as well as woody forest regrowth [29]. Apart from widespread persecution of one carnivorous mammal, the dingo (*Canis dingo*), hunting of vertebrates by humans has been negligible throughout the region for at least several decades.

**Fig 1:**
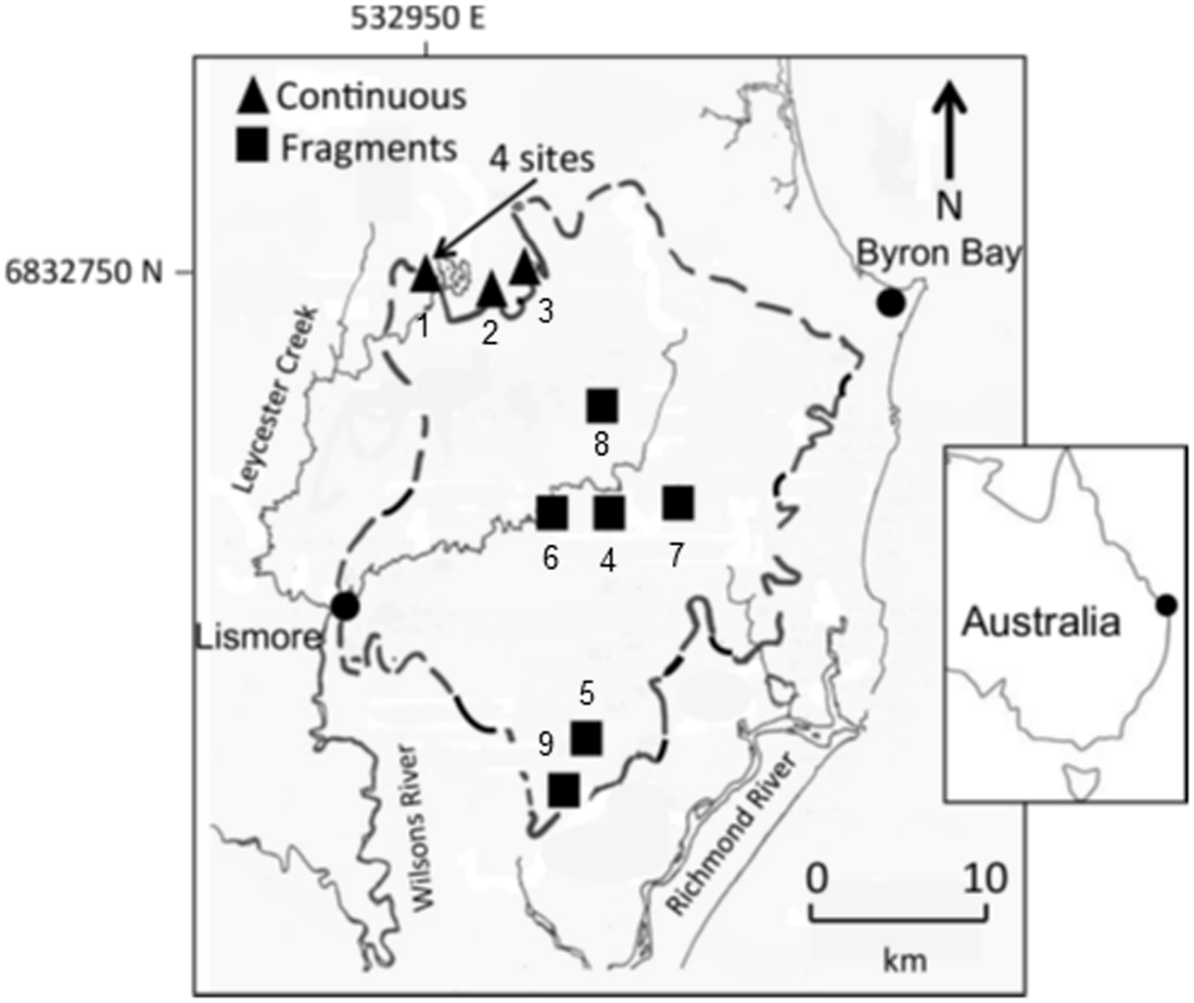
Study site locations and major water courses. Triangles represent continuous forest sites, squares represent fragments. Numbers correspond with site numbers in S1 Table. Solid lines represent water courses. Dashed line indicates boundary of the Big Scrub rainforest prior to European settlement [27].

Data were collected at 12 study sites, all on lowland basaltic soils, spread throughout the region (Fig 1; S1 Table): six in continuous forest (rainforest patches > 75 ha within the larger northern forest mosaic > 1000 ha) and six in fragments (3–21 ha). Each study site covered an area of approximately 100 m × 100 m (1 ha). The continuous forest sites were located in extensive conservation reserves at the region’s northern end. Four continuous sites situated in one area were at least 500 m apart while the other two were each > 4.0 km from all other sites (Fig 1). Fragments were located 4–10 km apart within the agricultural landscape and 7–29 km from the continuous rainforest areas to the north. Sites were selected based on a previous regional assessment of environmental characteristics together with floristic surveys which had been conducted to standardise soil type, elevation and vegetation type among sites.

### Field data collection

We established seed stations using a modification of similar methods used by Galetti et al. [5] and Fleury and Galetti [18] at each site, using seeds of 20 tree species known from previous floristic surveys to occur commonly across the study sites. These comprised nine large-seeded (width ≥ 10 mm) and 11 small-seeded (width < 10 mm.) species. A seed station for a given species consisted of five seeds together with a similar-sized artificial seed constructed from brown plasticine and tethered by 50 cm of nylon fishing line to a central metal peg. Plasticine seeds were used as part of an associated study investigating patterns of interaction between seeds and seed predators in the same study sites [30]. An independent samples t-test conducted among sites found no significant difference (*t* = 0.91, *P* = 0.37, df = 22) when comparing overall predation rates on actual seeds (not including plasticine) vs actual and plasticine seeds. Thus, plasticine seed predation was not considered separate from the actual seeds for this study. Two stations for each seed species were established simultaneously at each site where they were then exposed for five days. After five days’ exposure, the fate of each seed was recorded as either: intact (no detectable evidence of any physical interference); removed (seed completely destroyed or disappeared from its glued-on tether); or damaged (evidence of physical interference, such as teeth marks on the seed coat).

Seed stations were deployed during two field seasons, September-December 2013 and June-August 2015, with both the timing and duration of data collection a result of logistical considerations. During a field season, all seeds within each species were exposed at similar times in sites from both continuous forest and fragments. At any one time during field sampling, seed stations of 2–4 species (with two stations per species, per site) were exposed across the site network, with different seed species then being used in succession at particular stations (i.e. 24 stations per species across the 12 sites). Stations in concurrent use within any site were positioned >50 m apart and situated away from treefall gaps, paths and any other visibly disturbed areas.

In the first season, ten seed species were used (*Acmena ingens*, *Castanospora alphandii*, *Cryptocarya glaucescens*, *Cryptocarya obovata*, *Ehretia acuminata*, *Guioa semiglauca*, *Mallotus philippensis*, *Neolitsea dealbata Tabernaemontana pandacaqui*, *Wilkiea huegeliana*). In the second season, a further ten species were used (*Acmena hemilampra*, *Atractocarpus chartaceus*, *Brachychiton acerifolius*, *Castanospermum australe*, *Cinnamomum camphora*, *Cryptocarya glaucescens*, *Cryptocarya microneura*, *Denhamia celastroides*, *Ehretia acuminata*, *Eupomatia laurina*, *Melia azedarach*, *Podocarpus elatus*), together with two species (*E*. *acuminata*, *C*. *glaucescens*) from the previous season that were redeployed in the second season to enable tests of between-year variability [30]. Our overall study design thus comprised 12 sites (6 continuous forest, 6 fragments), 20 seed species, and two stations per species per site (four for the two species that were used twice), yielding a total of 44 stations per site across all species (with 528 stations and 3168 deployed seeds in total) over both seasons.

To record the vertebrate seed predators and their behaviour, we used automated motion-activated infra-red video cameras (Ltl Acorn 5210A 12MP or Scout Guard TC2201NA). At each site, one camera was positioned to continuously focus on one of the two seed stations per seed species for the five days during which the station was exposed. Consequently, ten camera sessions per site were recorded during the first field season (one per each of the ten deployed seed species) and twelve camera sessions were recorded during the second field season (one per each of the ten further deployed seed species and the two redeployed species). In the second season only, six stations without seeds were also monitored using cameras at each site to examine whether the presence of seeds in stations influenced the presence of vertebrate predators. This produced an overall total of 28 five-day camera sessions at each site, and 140 camera days in total (where each camera day is 24 hours) comprising 110 days at stations with seeds and 30 days at stations without seeds.

Camera footage was viewed to identify vertebrate animals to species where possible, also using independent information about local distributions from a variety of sources to clarify the suite of potential species. Species could be assigned in all cases except for a residual grouped category of unidentified species in the genus *Rattus*, comprising cases where no distinguishing featured were visible in the camera footage. On average, 294 events per site occurred that activated the cameras’ motion sensors, of which 92% were caused by movements of vertebrate animals, the remainder comprising wind-blown movements of vegetation.

To investigate the vertebrate species’ behavioural interactions with seeds, three categories of interest in seeds were defined for each footage sequence in which an individual animal was recorded at a seed station. Destructive interest occurred when a seed was bitten or pecked. Non-destructive interest involved an individual showing interest in seeds (for example, by approaching seeds and visually inspecting or sniffing them) but without physical interference. No interest occurred when an individual showed no detectable response to seeds.

### Data summary and analysis

To provide an index of vertebrate abundance at each site, we calculated the percent of total camera days (140/site) during which each identified species or predator functional group (see below) was present at each site, henceforth termed the sampling rate (Table 1), and also interpreted to indicate relative abundance. To assess how strongly the presence of seeds at stations influenced the presence of all recorded vertebrate species, we also separately calculated similar site-specific values for cases with and without seeds, for each predator taxon (termed % presence; Table 1). To quantify how each predator species and functional group interacted behaviourally with seeds at each sites, we calculated the percent of camera days on which it was recorded where it also showed either destructive interest or non-destructive interest in seeds (Table 1).

**Table 1:** Indices of relative abundance and behavioural interactions with seeds, from camera data.

Name of index	Measurement at each site	Mean
Sampling rate	Days recorded / total days (140).	a
% presence with seeds	Days recorded/ total days at stations with seeds (110).	a
% presence without seeds	Days recorded/ total days at stations without seeds (30).	a
Non-destructive interest	No. of days with non-destructive interest in seeds / total days recorded.	b
Destructive interest	No. of days with destructive interest in seeds / total days recorded.	b

“Days” refers to camera-days (each 24 hr). Indices were calculated for individual species and for functional groups of species.

“Mean” refers to site-specific values, further treated prior to analyses with species as replicates as follows: a = averaged across sites (all 12 sites or 6 sites per habitat) prior to analyses with species as replicates; b = averaged across sites where present (overall or within each habitat).

To classify each vertebrate species as a seed predator or not, we used both its destructive interest score and independent published information about its diet. A bird was considered to be a potential seed predator if it consumed seeds as part of its primary diet according to the description in the HANZAB series [31–34]. Potential mammalian seed predators were similarly derived from Strahan [35]. Guarino [36] was used for the single recorded reptile. To investigate fragmentation effects on functionally different subsets of seed predators, each species was also classified into a functional group based on its body size (small < 1.0 kg or large > 1.0 kg, [20], [21]) and class (bird or mammal) producing four groups in total: small bird, large bird, small mammal and large mammal.

Species-specific analyses of the patterns in vertebrate abundance and behaviour focused initially on 14 common identified vertebrate species (those recorded from >5 sites overall, irrespective of diet). The effect of seed provision on the percent presence of predator species was tested statistically by comparing percent presence values with seeds vs without seeds using a paired t-test, with the 14 species as replicates (and with presence values averaged across all 12 sites for each species). We used Pearson correlation analyses to test the strength of association between these percent presence values with and without seeds, across the same 14 predator species, and also to assess the association between predator species’ destructive and non-destructive interest scores, with the seven common seed predator species as replicates (in both cases using species-specific score values averaged across all 12 sites). The average precent presence values met parametric assumptions.

The pattern of among-site variation in community composition of common seed predator species was explored using two-dimensional nonmetric multidimensional scaling (NMDS) ordination [37], with Bray-Curtis between-site dissimilarities (N = 12 sites), using the ‘Vegan’ package, version 2.3–5, with 4,999 iterations in RStudio version 3.1.2 [38]. Biplot vectors were added to display individual taxa that were significantly (P < 0.05) associated with the ordination pattern. To test the statistical significance of differences in seed predator composition between fragments and continuous forest, analysis of similarities (ANOSIM) was used, also in the Vegan package. For each individual common seed predator species, and for each functional group, the effect of forest fragmentation on both its site sampling rate and its destructive interest score were statistically tested by comparing mean values in fragments with those in continuous forest, using sites as replicates (N = 6 per habitat) in independent-sample t-tests (a Mann-Whitney U test was used in cases where parametric assumptions were not met).

Independent measurements of seed predation at each site were obtained from the data on each seed’s physical fate at the end of the five-day exposure period. We used the percent of seeds that were interfered with (either damaged or removed) to indicate the overall rate of seed predation. Among all seeds that were interfered with, 95% were removed, and the site-specific average percentages interfered with and removed were highly correlated across sites (*r* = 0.92, *P* = 0.0001, df = 10 sites) and across seed species (*r* = 0.94, *P* = 0.0001, df = 18). Predation rates were calculated across all stations in a given site (including those with cameras present and absent), for each seed species, and collectively for all seed species, for large-seeded species, and for small-seeded species. The mean collective values of percent seed removal or damage in fragments were compared with the corresponding means in continuous forest using independent-sample t-tests, with sites as replicates (N = 6 per habitat), for all seeds, small seeds and large seeds. Finally, the site-specific scores of physical damage or removal for each seed species were averaged across all 12 sites, and the correspondence between these averages and the independent camera-based measurements of seed predation (percent camera days with destructive interest by any vertebrate seed predator towards each seed species, also averaged across sites) was assessed using Pearson correlation, with the 20 seed species as replicates.

Data values for various measurements, for site-species combinations and for functional groups, are provided in Parts A-G of S2 Table. All test statistic values for Pearson’s correlation (r) and t-tests were obtained using RStudio version 3.1.2 [35]. The threshold for statistical significance in all tests was *P* < 0.05.

### Ethics statement

This study was carried out under scientific licence SL101196 issued by the New South Wales Parks and Wildlife Service (NSWPWS), after reviewing all research procedures, including those used for observing animals. Animals were not physically handled and there was no spotlighting, and hence ethics approval was not required by the Griffith University Animal Ethics Committee. Names of authorities that approved fieldwork on each site are provided in S1 Table.

## Results

### Seed predator species composition and behavioural responses to seeds

Vertebrates were recorded on 56% (941 days) of all 1680 camera days. From the camera footage 18 taxa were identified to species level and four to different genera, giving a total of 22 identified taxa. These comprised nine mammals, 12 ground-dwelling birds and one reptile (Table 2). Three taxa identified to genus were subsequently attributed to the most likely species based on knowledge of the region’s rainforest fauna (Table 2). Particular species were recorded at 1–12 sites, with a minimum sampling rate of 0.1% of days (a single camera record) to 27% of days (for the large mammal *Thylogale* sp.–most probably *T*. *thetis*; Table 2). For all taxa recorded more than once, destructive interest scores > 0.10 corresponded with literature-based assessments (see Methods) that indicated potential seed predator roles (seeds being part of the primary diet). This yielded nine post-dispersal seed predator species, five mammals and four birds (Table 2), among which the generic *Rattus* sp. taxon probably comprised several species, including overlap with the two fully-identified *Rattus* species. Consequently, this taxon was excluded from species-specific analyses.

**Table 2:** Vertebrate taxa recorded by cameras, and their frequencies of occurrence and behavioural responses to seeds.

Group	Abbrev-iation	Taxon	Common name	Diet	Sampling rate		Response to seeds (% days)				Pred
					No. Sites	% days present^1^	% days with seeds^2^	% days w/o seeds^3^	Non-DI^4^	DI^5^	
SB	BACNO	*Accipiter novaehollandiae*	grey goshawk	IV; 5	1	0.1	0.1	0.0	0.00	0.00	N
SB	BCHIN	*Chalcophaps indica*	emerald dove	**S**F; 2	12	6.4	7.7	1.9	0.05	0.19	Y
SB	BCOLE	*Columba leucomela*	white-headed pigeon	**S**F; 2	1	0.1	0.1	0.0	0.00	0.00	Y
SB	BEOAU	*Eopsaltria australis*	eastern yellow robin	I; 3	10	0.8	0.9	0.3	0.00	0.00	N
SB	BLEME	*Leucosarcia melanoleuca*	wonga pigeon	**S**; 2	12	5.7	6.8	1.4	0.04	0.19	Y
SB	BORTE	*Orthonyx temminckii*	Australian logrunner	I; 3	8	1.1	1.4	0.0	0.00	0.00	N
SB	BPIVE	*Pitta versicolor*	noisy pitta	I; 4	12	4.2	5.2	0.6	0.02	0.00	N
SB	BPSOL	*Psophodes olivaceus*	eastern whipbird	I; 3	11	1.8	1.9	1.4	0.05	0.00	N
SB	BSECI	*Sericornis citreogularis*	yellow-throated scrubwren	I; 3	11	1.4	1.6	0.8	0.00	0.00	N
SB	BSECH	*Sericulus chrysocephalus*	regent bowerbird	F; 4	3	0.1	0.2	0.0	0.00	0.00	N
SB	BZOHE	*Zoothera heinei*	russet-tailed thrush	I; 4	2	0.2	0.0	0.0	0.00	0.00	N
LB	BALLA	*Alectura lathami*	Australian brush turkey	**S**FI; 5	12	11.0	12.3	5.8	0.03	0.38	Y
SM	MANSP	*Antechinus* sp.	Antechinus sp.	I; 6	4	0.8	1.0	0.0	0.00	0.00	N
SM	MMECE	*Melomys cervinipes*^6^	fawn-footed melomys	**S**FI; 6	6	1.3	1.6	0.0	0.10	0.30	Y
SM	MRAFU	*Rattus fuscipes*	bush rat	**S**FPI; 6	12	20.7	24.8	5.8	0.35	0.42	Y
SM	MRARA	*Rattus*	black rat	**S**FPI; 6	12	10.0	11.5	4.4	0.18	0.25	Y
SM	MRASP	*Rattus* sp.	rat sp.	**S**FPI; 6	12	7.2	8.5	2.5	0.14	0.13	Y
SM	MTAAC	*Tachyglossus aculeatus*	short-beaked echidna	I; 6	5	0.7	0.8	0.6	0.00	0.00	N
LM	MPENA	*Perameles nasuta*	long-nosed bandicoot	I; 6	12	2.4	3.0	0.3	0.06	0.00	N
LM	MTHSP	*Thylogale* sp.^7^	pademelon sp.	FP; 6	12	27.3	20.6	30.6	0.30	0.01	N
LM	MTRSP	*Trichosurus* sp.^8^	brushtail possum sp.	**S**FI; 6	12	19.5	19.7	18.6	0.29	0.26	Y
R	RVAVA	*Varanus varius*	lace monitor	IV; 1	3	0.2	0.2	0.0	0.00	0.00	N
Seed predator functional groups											
SB		Small bird			12	11.5	137	3.3	0.04	0.21	
LB		Large bird			12	11.0	12.3	5.8	0.03	0.38	
SM		Small mammal			12	33.5	39.2	12.5	0.31	0.41	
LM		Large mammal			12	19.5	19.7	18.6	0.27	0.26	
B		All birds			12	20.7	23.8	9.2	0.05	0.33	
M		All mammals			12	46.3	51.0	29.2	0.34	0.41	
ALL		All taxa			12	56.2	62.0	35.0	0.30	0.43	

‘Group’ refers to the functional group of each taxon: SB, small bird; LB, large bird SM, small mammal; LM, large mammal; R, reptile. ‘Diet’ is the primary diet of each taxon: S, seeds; F, fruit; P, vegetative plant parts; I, invertebrates; V, vertebrates; numbers indicate the sources of information on primary diet: 1, [36]; 2, [31]; 3, [32]; 4, [33]; 5, [34]; 6, [35]. ‘No. sites’ is the number of sites in which a taxon was recorded. ‘Pred’ indicates whether or not a given taxon was classified as a seed predator (Y, yes; N, no)

^1^% days present = average percentage of days a given taxon was recorded by cameras across 12 sites

^2^% days with seeds; average percentage of days in which a given taxon was recorded at cameras with seeds

^3^% days w/o seeds; average percentage of days in which a given taxon was recorded at cameras without seeds

^4^Non-DI = non-destructive interest; average (across sites) percentage of days in which a given taxon was recorded showing interest in seeds but not physically interfering with them, calculated from the total number of days in which it was recorded

^5^ DI = destructive interest; average (across sites) percentage of days in which a given taxon was recorded behaviourally interfering with seeds, calculated from the total number of days in which it was recorded

^6^*M*. *cervinipes* is the only know *Melomys* species known to occur in the rainforests of the study region [28], [39]

^7^Most likely *T*. *thetis* however may potentially include *T*. *stigmatica*

^8^Most likely *T*. *caninus* or *T*. *vulpecula*

A total of seven seed predator species were classified as common (present at > 5 sites; Table 2). These comprised three small mammals (all rodents; bush rat *Rattus fuscipes*, non-native black rat *Rattus rattus* and mosaic-tailed rat *Melomys cervinipes*), one large marsupial mammal (brushtail possum *Trichosurus* sp.), one large bird (Australian brush turkey *Alectura lathami*) and two small birds (both pigeons; emerald dove *Chalcophaps indica*, wonga pigeon *Leucosarcia melanoleuca*). All common predator species, except *M*. *cervinipes*, were recorded from all 12 study sites; overall sampling rates varied from 1.3% to 20.7% of camera days (respectively, for *M*. *cervinipes* and *R*. *fuscipes*; Table 2).

**Fig 2:**
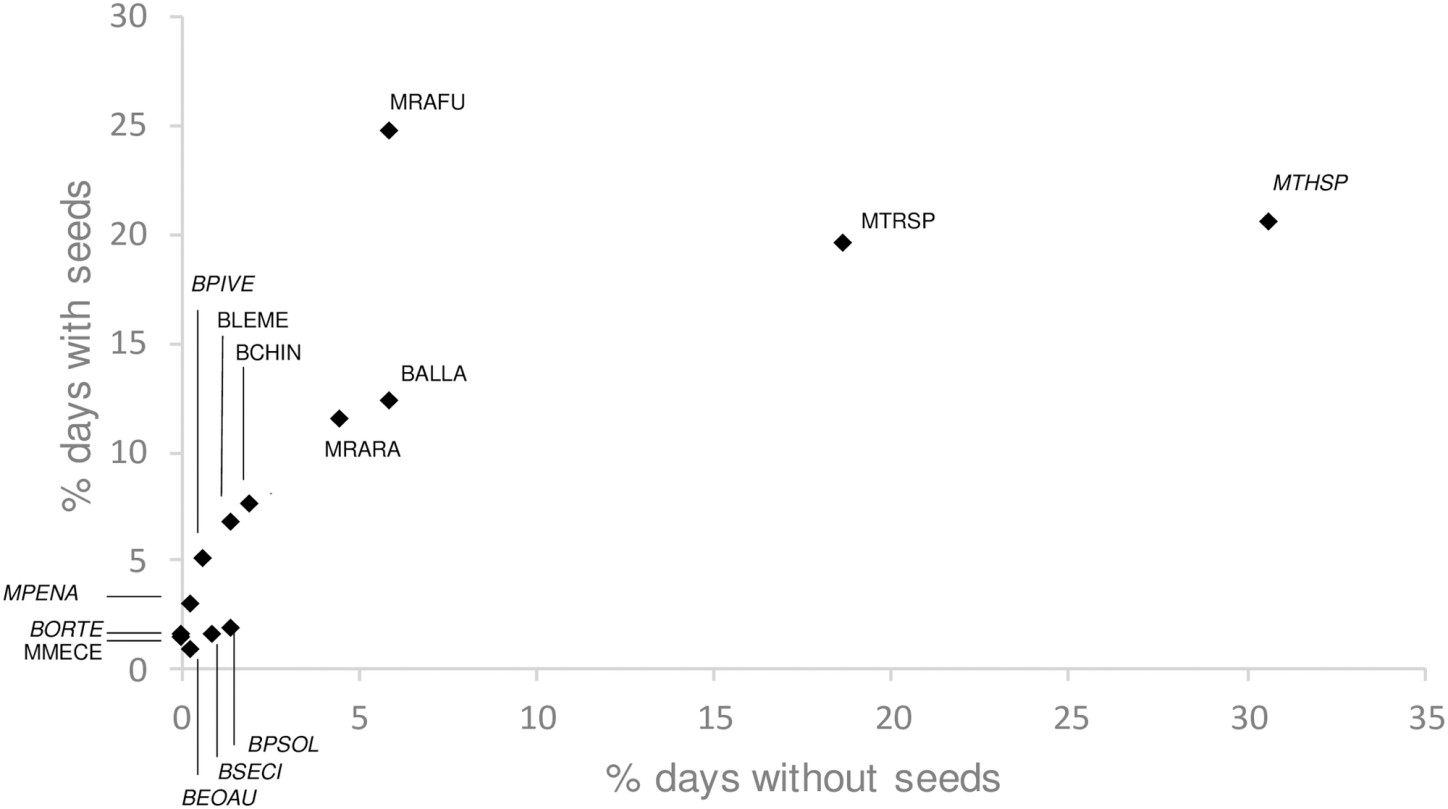
Effects of seeds on camera detection of all vertebrates. Points represent the 14 common identified species (see Table 2 for abbreviations). Italics show taxa not classified as seed predators. Axes show the percent of camera days (averaged across 12 sites) in which each vertebrate taxon was recorded, either with or without seeds present.

**Fig 3:**
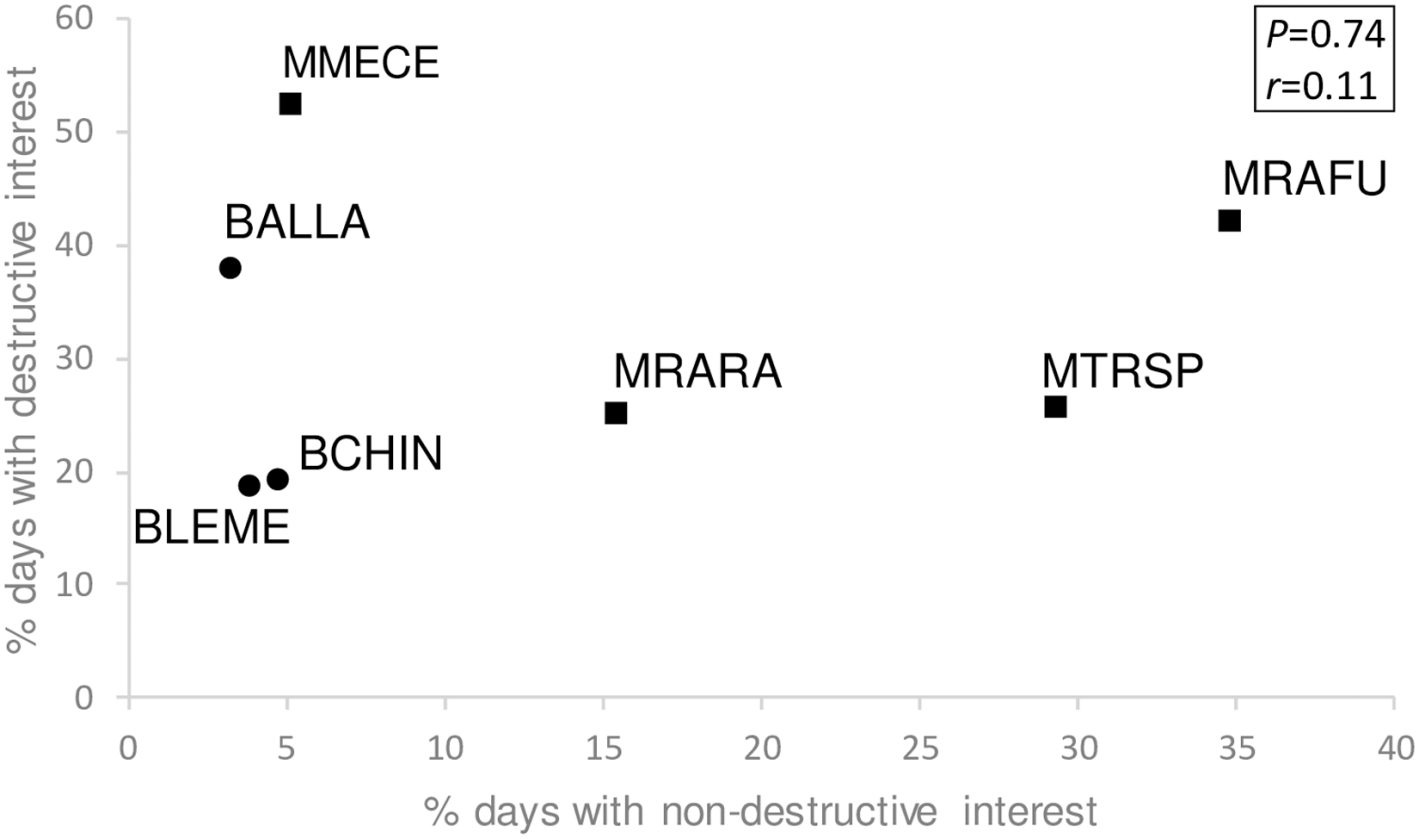
Behavioural interactions of common predator taxa with seeds. Squares represent mammals, circles represent birds (see Table 2 for abbreviations). Axes show the percent of camera days on which a predator was recorded (averaged across 12 sites) where it also showed non-destructive or destructive interest.

Across the 14 common identified vertebrate species, percent presence values were numerically higher with than without seeds for all seven seed predators and six of seven other-diet species (Table 2). Paired t-tests of percent presence values with versus without seeds indicated that this influence was statistically significant (*t* = 1.81, *P* = 0.04, df = 13 species), however this disappeared when *R*. *fuscipes* (which showed a distinctively strong presence response to seeds; Table 2, Fig 2) was excluded (*t* = 1.46, *P* = 0.08, df = 12). Both with and without *R*. *fuscipes*, percent presence values with versus without seeds were strongly positively correlated (respectively, *r* = 0.74, *P* = 0.003, df = 12, and *r* = 0.89, *P* = 0.0001, df = 11; Fig 2). Across the seven common seed predator species, levels of non-destructive and destructive interest both varied greatly and were not correlated with each other (*r* = 0.11, *P* = 0.74, df = 5; Fig 3). All mammals, except *M*. *cervinipes*, exhibited high levels of both non-destructive and destructive interest in seeds, while the bird that showed highest destructive interest (*A*. *lathami*) also showed low non-destructive interest (Fig 3).

### Effects of fragmentation on sampling rates and feeding behaviour

**Fig 4:**
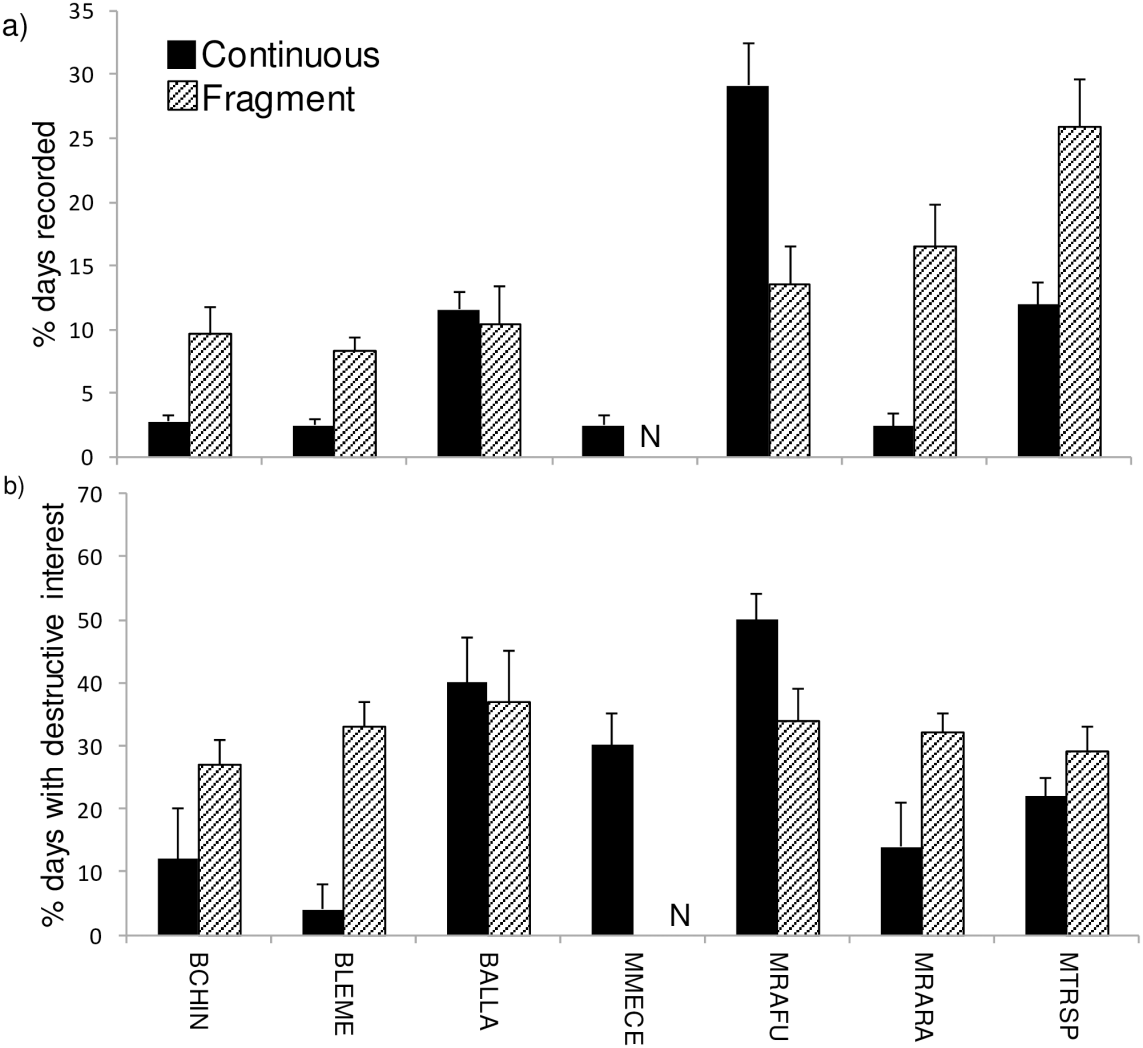
Effect of forest fragmentation on sampling rates and feeding behaviours of common seed predator taxa. See Table 2 for abbreviations (N = 6 sites in continuous forest, 6 in fragments; bars are SEs). a) Percent camera days that a taxon was recorded (sampling rate). b) Percent of these camera days in which there was physical interference with seeds (destructive interest). “N” refers to *M*. *cervinipes* not being recorded in fragments.

**Fig 5:**
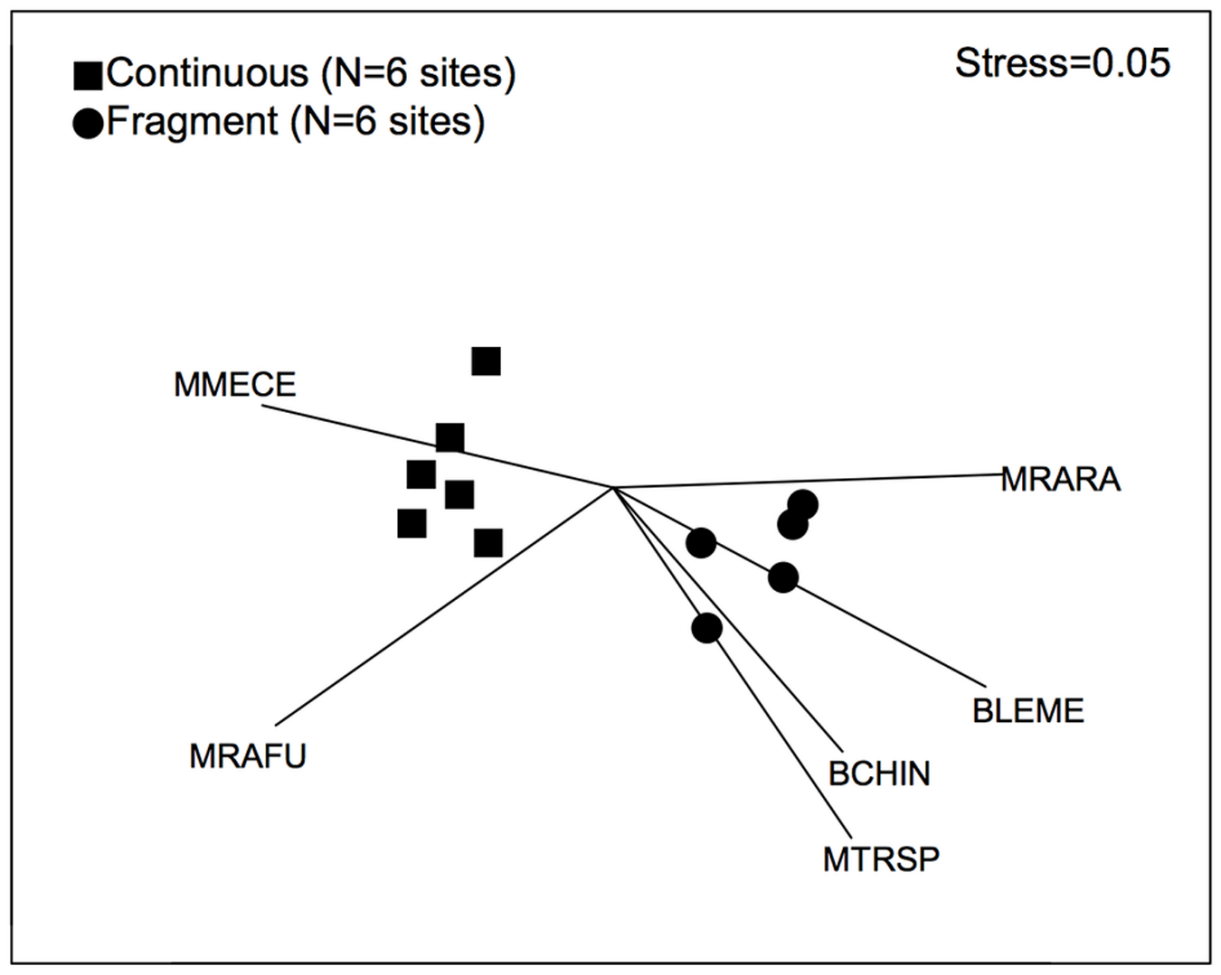
MDS ordination of among-site variation in seven common seed predator taxa. See Table 2 for abbreviations (N = 6 sites in continuous forest, 6 in fragments). Input data were the sampling rates (%days recorded) for each taxon. Lines show statistically significant biplot vectors indicating strength of each taxon’s association with different regions of the plot.

Sampling rates of most (six of seven) common seed predator species differed significantly between fragments and continuous forest, but their responses to fragmentation varied greatly (Table 3, Fig 4a). Among the three small mammals, *M*. *cervinipes* was confined to continuous forest while *R*. *fuscipes* was recorded twice as commonly in continuous forest as in fragments. This contrasted with *R*. *rattus*, which was much more common in fragments than in continuous forest. The large bird *A*. *lathami* had no detectable response to fragmentation whereas the remaining three species (two small birds and one large mammal) were all more common in fragments than in continuous forest (Table 3, Fig 4a). NMDS ordination (Fig 5) and ANOSIM (*R* = 0.88, *P* = 0.003) confirmed the large and distinct difference in community composition of common seed predators between fragments and continuous forest, with six species being significantly associated with the ordination pattern in a manner consistent with the species-specific analysis.

**Table 3:** Effect of habitat fragmentation (continuous forest *vs* fragments, N = 6 sited in each) on sampling rates of vertebrate seed predators, and on their feeding behaviours. See also Figs 4 and 6.

Type of seed predator	Sampling rate^1^			Destructive interest^2^		
	df	*t*^3^	*P*	df	*t*^3^	*P*
Individual species:						
* Chalcophaps indica*	10	N/A	**0.009**	10	-1.48	0.08
*Leucosarcia melanoleuca*	10	-4.51	**0.001**	10	-4.23	**0.002**
*Alectura lathami*	10	-0.0004	0.96	10	0.45	0.66
*Melomys cervinipes*^4^	10	N/A	0.001	N/A	N/A	N/A
*Rattus fuscipes*	10	5.92	**0.0004**	10	2.09	**0.03**
*Rattus rattus*	10	-4.22	**0.002**	10	-3.00	**0.02**
*Trichosurus sp*.	10	-3.00	**0.02**	10	-1.56	0.16
Functional groups:						
small birds	10	-4.20	**0.003**	10	-2.72	**0.02**
large birds	10	-0.0004	0.96	10	0.45	0.66
small mammals	10	1.83	0.10	10	2.51	**0.03**
large mammals	10	-3.00	**0.02**	10	-1.56	0.16
all birds	10	-2.15	**0.05**	10	-0.69	0.51
all mammals	10	-0.58	0.58	10	1.41	0.19
all predators	10	1.63	**0.05**	10	0.36	0.72

^1^Sampling rate (‘% days recorded’) is the average percent of days a given taxon was recorded by cameras in continuous forest and fragments (N = 6 sites in each)

^2^Destructive interest is the average percent of days in which a given taxon was recorded behaviourally interfering with seeds, calculated from the total number of days in which it was recorded in continuous forest and fragments (N = 6 sites in each).

^3^From independent-sample t-tests except for *C*. *indica* and *M*. *cervinipes* sampling rates, where Mann-Whitney U-tests were used due to unequal variances; *P* values are bolded if statistically significant (*P* < 0.05).

^4^*M*. *cervinipes* was absent from all fragments, hence destructive interest could not be calculated.

**Fig 6:**
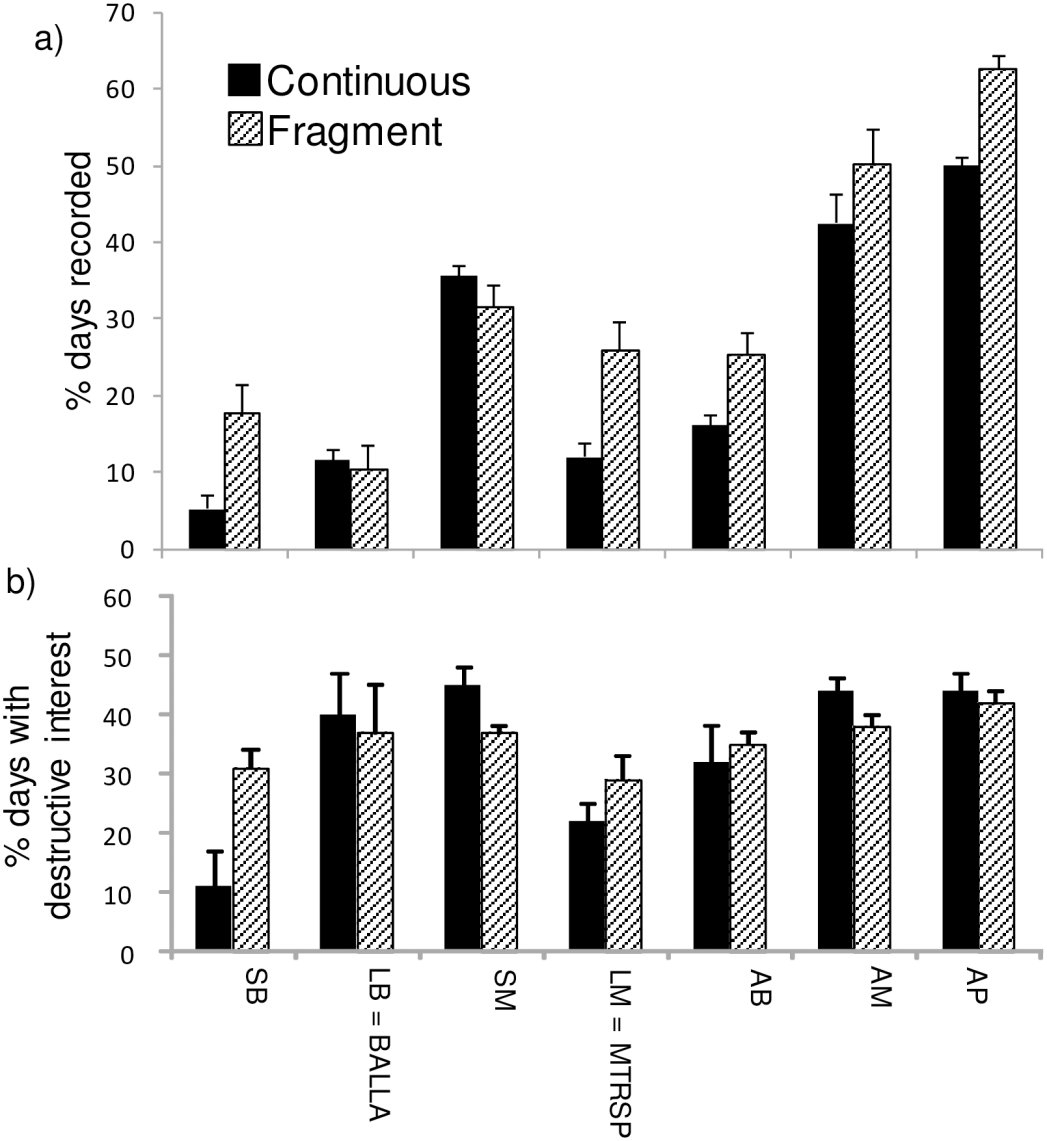
Effect of forest fragmentation on abundance and feeding behaviour of common seed predator functional groups. See Table 2 for abbreviations, Table 3 for statistical tests (N = 6 sites in continuous forest, 6 in fragments; bars are SEs). a) Percent camera days on which a functional group was recorded (sampling rate). b) Percent of these camera days on which a functional group physically interfered with seeds (destructive interest).

The level of destructive behaviour towards seeds that was exhibited by individual taxa also differed between continuous forest and fragments. One species, *R*. *fuscipes* (MRAFU) showed substantially higher levels of destructive interest in seeds in continuous forest than in fragments (Table 3, Fig 4b). Conversely, two other species showed much higher levels of destructive interest in seeds in fragments: *L*. *melanoleuca* (BLEME) and *R*. *rattus* (MRARA) (Table 3, Fig 4b). With respect to functional groups of vertebrate seed predators, small birds collectively were significantly more common in fragments than continuous forest, as was the case for all birds combined, and also for combined percent presence across the entire seed predator community (Table 3, Fig 6a). Destructive interest in seeds by small birds also increased in fragments, being almost three times greater than in continuous forest, whereas destructive interest by small mammals was less in fragments than in continuous forest. Other functional groups showed no significant responses (Table 3, Fig 6b).

Site-specific variation in rainforest patch size did not further account for the effects of fragmentation on either sampling rates of seed predator species and functional groups or their feeding behaviours. Both of these measurements were associated mainly with the dichotomy between the small fragments (< 25 ha in cleared matrix) and larger forest patches (>50 ha of rainforest embedded in drier forest types; S3 Table and Parts A and B of S1 Fig).

### Levels of physical interference and effects of fragmentation on rates of seed predation

**Fig 7:**
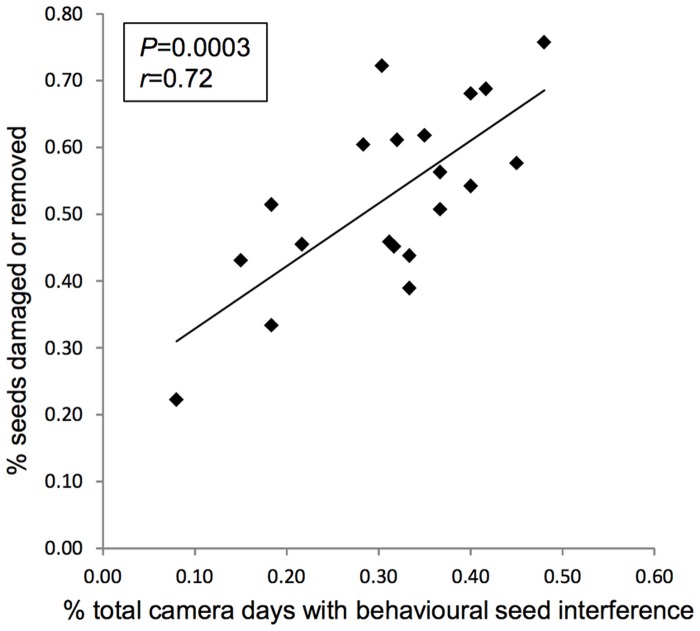
Relationship between behavioural seed interference events recorded by cameras (% total camera days with interference behaviour) and the physical fate of seeds at stations (% seeds damaged or removed). Points represent individual seed species (N = 20); each represented by the average values across 12 sites.

**Fig 8:**
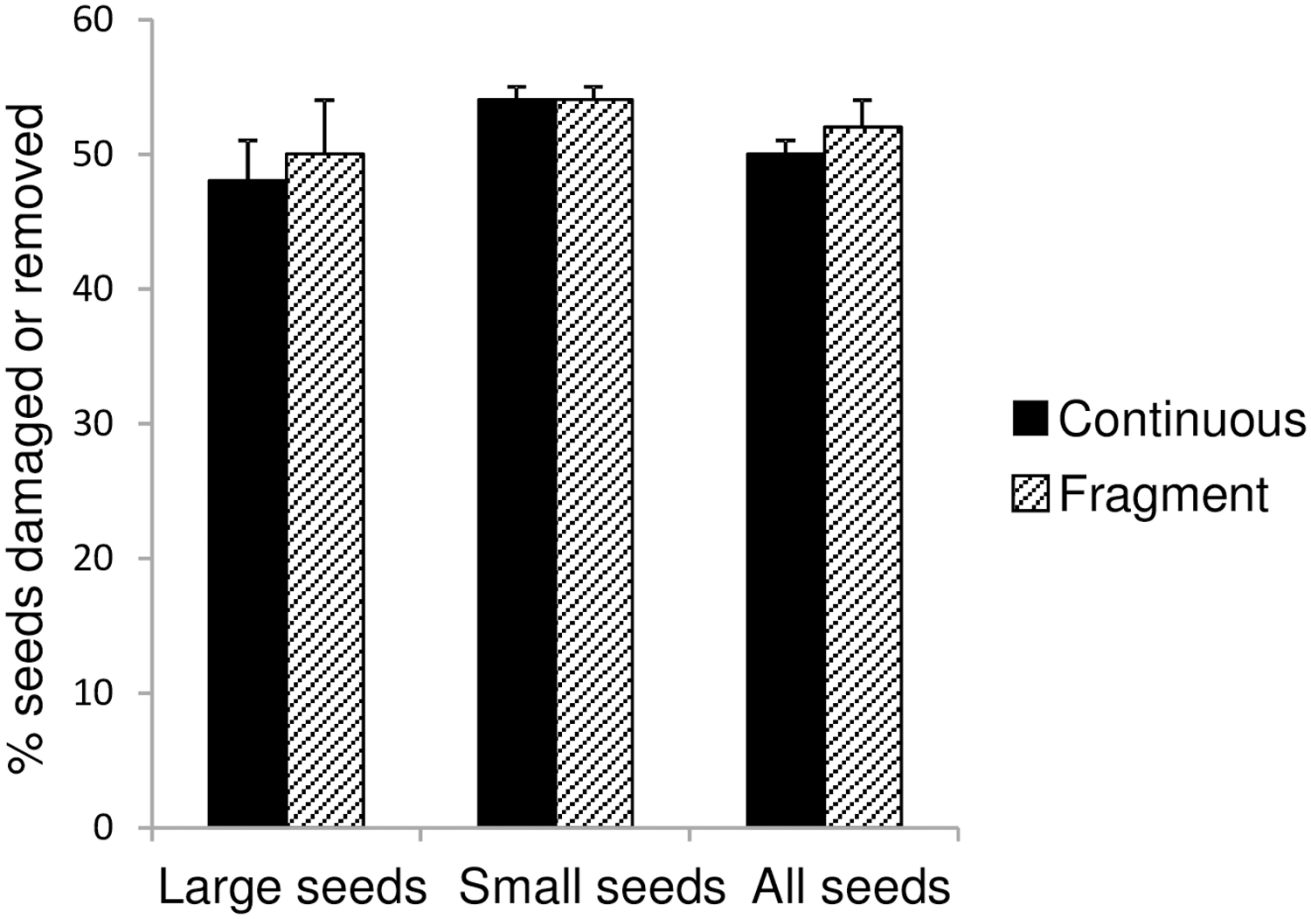
Effect of fragmentation on the physical fate of seeds at stations (% damaged or removed). N = 6 sites in continuous forest, 6 in fragments; bars are SEs. Large seeds were ≥ 10mm width, small seeds < 10mm.

The level of behavioural interference recorded by cameras was strongly correlated with the percent of deployed seeds which had been physically removed or damaged after five days of exposure (*r* = 0.72, *P* = 0.0003, df = 18 species; Fig 7). Despite weak trends for greater removal or damage in fragments to large seeds and to all seeds (Fig 8), t-tests comparing the two habitats showed no significant differences (large seeds *t* = -1.01, *P* = 0.44; small seeds *t* = 0.06, *P* = 0.95; all seeds *t* = -1.35, *P* = 0.13; N = 6 sites per habitat).

## Discussion

We identified four mammal and three bird species as significant post-dispersal seed predators in this subtropical Australian rainforest. Habitat fragmentation substantially altered the species composition of this seed predator community. Furthermore, differences in levels of destructive interest in seeds shown by individual species were also found in association with fragmentation, whose magnitude generally increased in each species’ habitat of greater abundance. Since reduced abundance of some species in fragments was offset by increases in others, there were no substantial net differences in overall rates of seed removal or damage between continuous and fragmented habitats.

### Seed predator occurrence and feeding behaviour

All three identified rodent species (*R*. *fuscipes*, *M*. *cervinipes*, *R*. *rattus*; all family Muridae) functioned as important post-dispersal seed predators in the study region. Murid rodents have also been identified as significant post-dispersal predators of rainforest seeds in the neotropics [8], Africa [9] and tropical Australia [7]. Rodents from other families can also have this role. For example, in the neotropics Sanchez-Cordero and Martinez-Gallardo [40] identified *Heteromys desmarestianus* (family Heteromyidae) and *Peromyscus mexicanus* (family Cricetidae) as major seed predators of two tree species in Mexico while Iob and Vieira [10] found that cricetid rodents were the main seed predators of the tree *Araucaria angustifolia* in Brasil.

Ground-active granivorous bird species were also significant contributors to post-dispersal seed predation in the Big Scrub. The large, mainly terrestrial bird *A*. *lathami* (family Megapodiidae), whose diet comprises seeds together with fruits and insects [34], together with two small pigeons (*C*. *indica*, *L*. *melanoleuca*; family Columbidae), which spend some of their time in above-ground vegetation but mainly feed on fallen seeds [31], were identified as important post-dispersal seed predators. A third pigeon species, *Columba leucomela*, which was recorded only once by this study’s ground-focused cameras, is a common arboreal pre-dispersal seed predator in Australian subtropical rainforest [41]. Birds have rarely been targeted as potentially important seed predators in ecological studies of seed fate in rainforests. However, Christianini and Galetti [42] concluded from survey and experimental exclosure data in neotropical rainforest that pigeons contributed significantly to rates of seed predation, together with the large ground-dwelling tinamou (family Tinamidae). At a neotropical rainforest site, where the abundance of larger-bodied (> 2 kg) mammalian seed predators had been greatly reduced by hunting, Pizo and Vieira [25] identified pigeons as the major seed predators. We expect that further studies in other regions using seed stations with automated cameras will further reveal a diversity of other birds that are functionally important as post-dispersal seed predators.

None of the seven vertebrate taxa identified as common seed predators in this study are commonly known to secondarily disperse seeds as a result of caching or hoarding behaviour. Seed hoarding by murid rodents has been considered negligible in studies in rainforests of southern Asia [43] and northern Australia [44], where seed removal was also interpreted as seed predation. Likewise, studies in the neotropics have identified murid and critecid rodents as major seed predators, and interpreted seed removal as predation [5], [22].

In the present study, *R*. *fuscipes* was the only species to show a strong micro-spatial attraction to seeds, being recorded much more frequently when seeds were present than when they were absent. When in the vicinity of seeds, most common mammal seed predators showed relatively high levels of both non-destructive and destructive interest, whereas the birds had low non-destructive interest, even when their destructive interest was high. The mammals were all recorded at night and all are nocturnal feeders [35] that would closely approach seeds for olfactory or other cues to assess their suitability as food. In contrast, the birds were all recorded by cameras in daylight, are intrinsically diurnal [31], [34] and could visually assess seeds from a distance beyond camera range before approaching them for consumption. Camera-based data on direct handling of seeds therefore appear to be the most useful indicators of predatory intent. This inference is supported by the strong observed correlation across seed taxa between rates of destructive behaviour towards seeds and the seeds’ actual independently recorded physical fates. Although most seed taxa in this comparison were deployed in a single year, the predation rates were temporally consistent in the two species that were deployed in both years [30], indicating that single-year sampling of other seed species would be unlikely to have affected this result.

### Effects of fragmentation on abundance of ground-active vertebrates

The abundance responses of ground active post-dispersal seed predators to habitat fragmentation varied greatly, with some species affected negatively while others were affected positively. Even within the rodent family Muridae, native species declined (*R*. *fuscipes*, *M*. *cervinipes*) while the invasive species (*R*. *rattus*) increased in fragments. The two small common pigeons and the larger mammal *Trichosurus* sp. all increased in fragments. Thus, across the whole post-dispersal vertebrate seed predator community, the main effect of forest fragmentation was an altered composition of species and functional groups rather than an overall increase or decrease in the abundance of seed predators. All seed predators collectively were significantly more common in fragments, but only by about 20% of the value in continuous forest. This variety of species-specific responses to rainforest fragmentation within a single community is mirrored by the varied nature of responses reported for individual mammalian seed predator species studied in other regions, including both decreases [9], [20], [23]and increases [21], [22] in abundance within fragments.

Among the Australian rodents, trapping studies have reported fragmentation-associated declines in *M*. *cervinipes*, coupled with increased abundance of *R*. *rattus*, both in the Big Scrub [39], [45] and in other geographically separate regions to its north [46] and south [47], [48]. This consistency with the present study’s findings supports our inference that the observed differences between fragments and continuous forest were due to habitat fragmentation rather than to the differing within-region spatial locations of the two habitats (Fig 1). For one rodent species, *R*. *fuscipes* (the most common and significant seed predator), the reported effects of fragmentation have varied among studies. In other subtropical regions, Dunstan and Fox [47] found that the abundance of *R*. *fuscipes* decreased in small isolated patches whereas Bentley et al. [46] found no difference between fragments and continuous forest. Trap-based studies in the Big Scrub region during 1991–2005 (and using some of the same sites as this study) have previously reported that *R*. *fuscipes* was absent from most fragments but abundant in continuous forest [39], [45], whereas the present study recorded *R*. *fuscipes* in all fragments, albeit at lower abundance than in continuous forest. This apparent increase may potentially reflect habitat restoration works that have been conducted in the Big Scrub fragments since the late 1990s [29]. Additionally, functional habitat linkages are likely to have concurrently improved, due to a substantial increase in woody vegetation cover across the landscape [29], [49], [50], comprising both unassisted forest regrowth dominated by the non-native tree *Cinnamomum camphora*, and extensive development of macadamia plantations (whose nuts are eaten by rodents). Such local and landscape-scale changes may have supported recent colonisation by, or population increases of, *R*. *fuscipes* in the Big Scrub’s rainforest fragments.

For birds, various studies of responses to fragmentation in ground-active and understorey species (irrespective of diet) have reported reduced abundances in neotropical rainforest fragments [51], [52]. However, Pizo and Vieira [25] found that five terrestrial seed-eating bird species (four pigeons and a tinamou) increased in abundance in a Brazilian rainforest fragment. The present study likewise found that small seed-eating birds were collectively more common in fragments than in continuous forest, and small insectivorous birds showed a similar pattern of higher abundance in fragments (Part H of S2 Table). The current abundance of small birds in the Big Scrub’s habitat fragments may reflect a recently-improved habitat quality and landscape connectivity (as for *R*. *fuscipes*). Moran et al. [41] found that one of the seed predators (*C*. *indica*) did not differ in abundance between continuous rainforest and fragments in a more northern Australian subtropical region where the fragments had not been the subject of restoration works. Greater population persistence of both insectivorous and omnivorous understorey bird species fragments has been associated with greater habitat connectivity in Brasilian rainforest [53]. These various findings collectively indicate that, as for mammals, individual avian seed predator species are likely to display both positive and negative responses to fragmentation of rainforest habitat, depending on both species-specific and habitat-related factors.

### Cascading effects of fragmentation on seed predation and tree regeneration

In the present study, feeding by all ground-active vertebrates resulted in high rates of seed damage and removal (about 50% after five days), with a consequently high potential to influence seedling recruitment. The impact of feeding by ground-active seed predator species on mortality rates of fallen seeds depends on both the abundance of predators and their feeding behaviours. If either of these changes in habitat fragments, then altered rates of seedling recruitment are likely to follow. Previous studies have targeted the abundance responses of particular seed predators for investigation, together with the implications for selected tree species. For example, in neotropical rainforest fragments, depleted populations of cricetid rodents were associated with reduced predation on seeds of one studied tree species [20]. Also in neotropical fragments, Guariguata et al. [21] found higher predation rates on seeds of two studied tree species, associated with population increases in small (<1000 g) heteromyid rodents, which in turn were attributed to reduced abundance of larger (> 30 kg) mammalian carnivores in fragments. However, conclusions of both these studies were based on indirect evidence of predator-seed interactions.

The present study’s camera-based data showed that a species’ level of destructive interest in seeds often increased in its habitat of greater abundance. For example, in fragments the pigeon *L*. *melanoleuca* occurred more commonly and also showed a higher destructive interest, whereas the rodent *R*. *fuscipes* occurred less commonly, with a lower destructive interest. Fragmentation-related changes in this seed-eating community could therefore strongly influence patterns of tree regeneration because changes in abundance and feeding behaviour reinforced each other. The processes that underlie fragmentation-associated changes in feeding behaviour are unclear. One possibility is that greater abundance leads to higher intraspecific competition for food and therefore a more rapid and intense response to the provision of seeds. Fluctuations in background levels of seed availability can also lead to variability in patterns of feeding behaviour, for example if large seed crops are produced by any tree species on-site, predator satiation [54] could reduce the rates at which target seeds are removed. However, any fluctuations in background levels of seed production are unlikely to have influenced the findings of the present study, because we used multiple replicate sites in fragments and continuous forest, and sampled both habitats simultaneously. Such fluctuations may have contributed to among-site variability but did not prevent the study from detecting the observed effects of fragmentation.

More generally, if functionally different subsets of seed predators are affected in different ways by habitat fragmentation, the impacts will likely differ among tree species. For example, in one neotropical rainforest study, the average mass of seeds surviving to germination was greater where hunting had depleted the abundance of large (> 1000 g) seed predators than in areas protected from hunting [55]. The seed predator communities in this study’s rainforest fragments were characterised by increased collective abundances of both small birds and larger mammals together with unchanged collective abundances of large birds and small mammals. Additionally, since fragmentation-associated decreases in some seed predator species were offset by increases in others, fragments and continuous forest had similar overall rates of physical seed removal or damage when calculated over multiple tree species. Therefore, any changes in tree recruitment that occur as a consequence of fragmentation-induced changes in the vertebrate seed predators would likely be complex and dependent on the amount of functional substitution in the interaction networks that link the various individual predator and seed species. Our findings further suggest that management actions aimed at improving habitat quality in fragments and the surrounding matrix could at least partially reverse such changes in tree recruitment by facilitating recovery of the vertebrate seed predator community. Conducting community-wide assessments of the effects of anthropogenic disturbances on predator-prey networks with seeds as subjects is a fertile ground for future study in many regions.

## Supporting information

S1 Table Study site information. Site type in parantheses (c, continuous; f, fragments). Rainforest patch size refers to the area of rainforest habitat, which is surrounded by pasture in the case of fragments and by drier eucalypt forest in the case of continuous forest.
(DOCX)

S2 Table **Part A. Sampling rates of vertebrate taxa and predator functional groups.** Calculated as the percentage of days a given taxon was recorded by cameras with and without seeds (140 camera days/site), averaged across continuous forest and fragments (N = 6 in each) and all sites (N = 12). Standard errors in parentheses. **Part B. Average percentage of days a given taxon was recorded by cameras with seeds (110 camera days/site).** Averaged across continuous forest and fragments (N = 6 in each) and all sites (N = 12). Standard errors in parentheses. **Part C. Average percentage of days a given taxon was recorded by cameras without seeds (30 camera days/site).** Averaged across continuous forest and fragments (N = 6 in each) and all sites (N = 12). Standard errors in parentheses. **Part D. Non-destructive interest scores of vertebrate taxa and predator functional groups.** Calculated as the percent of camera days a given taxon was recorded where it showed interest in seeds without physically interfering with them, averaged across the number of sites where recorded. ‘N’ represents vertebrate not recorded at a site. Standard errors in parentheses. **Part E. Destructive interest scores of vertebrate taxa and predator functional groups.** Calculated as the percent of camera days a given taxon was recorded where it physically interfered with seeds, averaged across the number of sites where recorded. ‘N’ represents vertebrate not recorded at a site. Standard errors in parentheses. **Part F. Proportion of seeds removed or damaged after five days exposure at seed stations, for each seed species.** Averaged across 12 sites, with 10 seeds per site for each (20 seeds for asterisked species), a total of 120 seeds per species (240 if asterisked). L large (>10 mm), S small (<10 mm). **Part G. Proportion of seeds removed or damaged after five days of exposure at seed stations, for each site.** Averaged across measurements for each of 20 seed species at each site (derived from 12 seeds/site for each of 18 species and 24 seeds/site for two other species, totalling 6,336 seeds overall). Standard errors in brackets. **Part H. Sampling rates of small insectivorous birds.** Calculated as the percentage of days a given taxon was recorded by cameras with and without seeds (140 camera days/site), averaged across continuous forest and fragments (N = 6 in each) and all sites (N = 12). Standard errors in parentheses.
(DOCX)

S3 Table Effect of rainforest patch size on sampling rates and feeding behaviours of seed predator species and functional groups. Rainforest patch size is the area of rainforest habitat, which is surrounded by pasture in the case of fragments and by drier eucalypt forest in the case of continuous forest.
(DOCX)

S1 Fig **Part A. Effect of rainforest patch size on vertebrate seed predator abundance.** Abundance calculated as the percent camera days that a taxon was recorded (sampling rate) at a given site. Circles represent fragments, squares represent continuous forest (N = 6 in each). Continuous rainforest sites are contiguous with extensive drier eucalypt forest, while fragments are surrounded by pasture. **Part B. Effect of rainforest patch size on vertebrate seed predator feeding behaviour.** Feeding behaviour calculated as the percent camera days that a taxon was recorded physically interfering with seeds (destructive interest), calculated from the total number of days in which it was recorded at a given site. Circles represent fragments, squares represent continuous forest (N = 6 in each). Continuous rainforest sites are contiguous with extensive drier eucalypt forest, while fragments are surrounded by pasture.
(DOCX)
