# Supplementary material for: Impacts of rainforest fragmentation on the composition of ground-active vertebrate communities and their patterns of seed consumption
Source: PLoS One. 2018 Sep 12;13(9):e0202870. doi: 10.1371/journal.pone.0202870 (PMC6135387; doi:10.1371/journal.pone.0202870)
Supplement: S1 Table — Site type in parantheses (c, continuous; f, fragments). Rainforest patch size refers to the area of rainforest habitat, which is surrounded by pasture in the case of fragments and by drier eucalypt forest in the case of continuous forest. (DOCX) [file pone.0202870.s001.docx]

**S1 Supporting information**

**S1 Table. Study site information.**

| **Site no.** | **Site name** | **Rainforest patch size (ha)** | **Habitat type** | **Coordinates** | **Authority responsible for allowing access** |
| --- | --- | --- | --- | --- | --- |
| 1^*^ | Big Scrub 1 (c) | 148 | Continuous | 28⁰38’17”S 153⁰19’48”E | New South Wales Parks and Wildlife Service |
| 1^*^ | Big Scrub 2 (c) | 148 | Continuous | 28⁰38’00”S 153⁰19’47”E | New South Wales Parks and Wildlife Service |
| 1^*^ | Big Scrub 3 (c) | 148 | Continuous | 28⁰37’57”S 153⁰20’07”E | New South Wales Parks and Wildlife Service |
| 1^*^ | Big Scrub 4 (c) | 148 | Continuous | 28⁰38’18”S 153⁰20’08”E | New South Wales Parks and Wildlife Service |
| 2 | Boomerang Falls (c) | 76 | Continuous | 28⁰38’10”S 153⁰22’47”E | New South Wales Parks and Wildlife Service |
| 3 | Minyon Falls (c) | 82 | Continuous | 28⁰36’46”S 153⁰23’30”E | New South Wales Parks and Wildlife Service |
| 4 | Booyong Flora Reserve (f) | 11.9 | Fragment | 28⁰44’36”S 153⁰26’53”E | Booyong Recreation Reserve Trust |
| 5 | Davis Scrub (f) | 10.5 | Fragment | 28⁰51’57”S 153⁰24’19”E | New South Wales Parks and Wildlife Service |
| 6 | Dawes Bush (f) | 6.6 | Fragment | 28⁰44’58”S 153⁰24’47”E | private land^#^ |
| 7 | Emery’s Scrub (f) | 3.7 | Fragment | 28⁰44’57”S 153⁰28’48”E | private land^#^ |
| 8 | Johnson’s Nature Reserve (f) | 21.5 | Fragment | 28⁰41’48”S 153⁰25’28”E | New South Wales Parks and Wildlife Service |
| 9 | Victoria Park (f) | 7.9 | Fragment | 28⁰54’12”S 153⁰24’41”E | New South Wales Parks and Wildlife Service |

^*^Four sites were all located within the one study area

^#^The owner of the land gave permission to conduct the study on this site.
