# Supplementary material for: Impacts of rainforest fragmentation on the composition of ground-active vertebrate communities and their patterns of seed consumption
Source: PLoS One. 2018 Sep 12;13(9):e0202870. doi: 10.1371/journal.pone.0202870 (PMC6135387; doi:10.1371/journal.pone.0202870)
Supplement: S2 Table — Part A. Sampling rates of vertebrate taxa and predator functional groups. Calculated as the percentage of days a given taxon was recorded by cameras with and without seeds (140 camera days/site), averaged across continuous forest and fragments (N = 6 in each) and all sites (N = 12). Standard errors in parentheses. Part B. Average percentage of days a given taxon was recorded by cameras with seeds (110 camera days/site). Averaged across continuous forest and fragments (N = 6 in each) and all sites (N = 12). Standard errors in parentheses. Part C. Average percentage of days a given taxon was recorded by cameras without seeds (30 camera days/site). Averaged across continuous forest and fragments (N = 6 in each) and all sites (N = 12). Standard errors in parentheses. Part D. Non-destructive interest scores of vertebrate taxa and predator functional groups. Calculated as the percent of camera days a given taxon was recorded where it showed interest in seeds without physically interfering with them, averaged across the number of sites where recorded. ‘N’ represents vertebrate not recorded at a site. Standard errors in parentheses. Part E. Destructive interest scores of vertebrate taxa and predator functional groups. Calculated as the percent of camera days a given taxon was recorded where it physically interfered with seeds, averaged across the number of sites where recorded. ‘N’ represents vertebrate not recorded at a site. Standard errors in parentheses. Part F. Proportion of seeds removed or damaged after five days exposure at seed stations, for each seed species. Averaged across 12 sites, with 10 seeds per site for each (20 seeds for asterisked species), a total of 120 seeds per species (240 if asterisked). L large (>10 mm), S small (<10 mm). Part G. Proportion of seeds removed or damaged after five days of exposure at seed stations, for each site. Averaged across measurements for each of 20 seed species at each site (derived from 12 seeds/site for each of 18 species [file pone.0202870.s002.docx]

**S2 Supporting information**

**S2 Table. Part A. Sampling rates of vertebrate taxa and predator functional groups.**

|  | Continuous forest | | | | | | | Fragments | | | | | | | | All sites |
| --- | --- | --- | --- | --- | --- | --- | --- | --- | --- | --- | --- | --- | --- | --- | --- | --- |
| Vertebrate taxa | 1 | 2 | 3 | 4 | 5 | 6 | **Av.** | 1 | 2 | 3 | 4 | 5 | 6 | **Av.** | **Av.** | |
| *Accipiter novaehollandiae* | 0.0 | 0.0 | 0.0 | 0.0 | 0.0 | 0.0 | **0.0 (0.0)** | 0.0 | 0.7 | 0.0 | 0.0 | 0.0 | 0.0 | **0.1 (0.1)** | **0.1 (0.1)** | |
| *Chalcophaps indica* | 1.4 | 2.9 | 3.6 | 4.3 | 1.4 | 2.9 | **2.7 (0.5)** | 19.3 | 7.1 | 5.0 | 13.6 | 6.4 | 9.3 | **10.1 (2.2)** | **6.4 (1.5)** | |
| *Columba leucomela* | 0.0 | 0.0 | 0.0 | 0.0 | 0.0 | 0.0 | **0.0 (0.0)** | 0.7 | 0.0 | 0.0 | 0.0 | 0.0 | 0.0 | **0.1 (0.1)** | **0.1 (0.1)** | |
| *Eopsaltria australis* | 0.7 | 0.7 | 0.7 | 0.0 | 0.7 | 0.0 | **0.5 (0.2)** | 0.7 | 1.4 | 1.4 | 1.4 | 1.4 | 0.0 | **1.1 (0.2)** | **0.8 (0.2)** | |
| *Leucosarcia melanoleuca* | 2.9 | 1.4 | 2.1 | 3.6 | 3.6 | 1.4 | **2.5 (0.4)** | 9.3 | 7.1 | 9.3 | 12.9 | 5.0 | 9.3 | **8.8 (1.1)** | **5.7 (1.1)** | |
| *Orthonyx temminckii* | 2.9 | 1.4 | 0.0 | 1.4 | 0.7 | 0.7 | **1.2 (0.4)** | 0.0 | 0.0 | 0.7 | 0.7 | 5.0 | 0.0 | **1.1 (0.8)** | **1.1 (0.4)** | |
| *Pitta versicolor* | 0.7 | 4.3 | 3.6 | 3.6 | 2.9 | 0.7 | **2.6 (0.6)** | 8.6 | 8.6 | 3.6 | 5.7 | 3.6 | 5.0 | **5.8 (0.9)** | **4.2 (0.7)** | |
| *Psophodes olivaceus* | 1.4 | 1.4 | 0.0 | 2.1 | 2.1 | 0.7 | **1.3 (0.3)** | 2.9 | 1.4 | 2.1 | 0.7 | 4.3 | 2.1 | **2.3 (0.5)** | **1.8 (0.3)** | |
| *Sericornis citreogularis* | 2.1 | 0.0 | 0.0 | 0.7 | 0.0 | 0.0 | **0.5 (0.4)** | 0.0 | 0.7 | 0.7 | 0.0 | 10.0 | 2.9 | **2.4 (1.6)** | **1.4 (0.8)** | |
| *Sericulus chrysocephalus* | 0.0 | 0.0 | 0.0 | 0.0 | 0.0 | 0.0 | **0.0 (0.0)** | 0.7 | 0.0 | 0.7 | 0.0 | 0.0 | 0.0 | **0.2 (0.2)** | **0.1 (0.1)** | |
| *Zoothera heinei* | 0.0 | 2.1 | 0.7 | 0.0 | 0.0 | 0.0 | **0.5 (0.4)** | 0.0 | 0.0 | 0.0 | 0.0 | 0.0 | 0.0 | **0.0 (0.0)** | **0.2 (0.2)** | |
| *Alectura lathami* | 12.9 | 5.7 | 10.0 | 12.1 | 14.3 | 14.3 | **11.5 (1.3)** | 5.7 | 1.4 | 5.0 | 14.3 | 20.0 | 15.7 | **10.4 (3.0)** | **11.0 (1.6)** | |
| *Antechinus sp.* | 0.0 | 5.0 | 2.1 | 1.4 | 0.0 | 0.7 | **1.5 (0.8)** | 0.0 | 0.0 | 0.0 | 0.0 | 0.0 | 0.0 | **0.0 (0.0)** | **0.8 (0.4)** | |
| *Melomys cervinipes*^1^ | 2.1 | 4.3 | 0.7 | 0.7 | 2.1 | 5.0 | **2.5 (0.7)** | 0.0 | 0.0 | 0.0 | 0.0 | 0.0 | 0.0 | **0.0 (0.0)** | **1.3 (0.5)** | |
| *Rattus fuscipes* | 17.1 | 28.6 | 22.9 | 39.3 | 34.3 | 32.9 | **29.2 (3.3)** | 24.3 | 2.1 | 7.9 | 12.9 | 16.4 | 10.0 | **12.3 (3.1)** | **20.7 (3.3)** | |
| *Rattus rattus* | 5.0 | 5.0 | 0.7 | 3.6 | 0.0 | 0.0 | **2.4 (1.0)** | 13.6 | 19.3 | 7.1 | 26.4 | 12.9 | 26.4 | **17.6 (3.2)** | **10.0 (2.8)** | |
| *Rattus sp.* | 0.7 | 6.4 | 7.9 | 9.3 | 7.9 | 5.7 | **6.3 (1.2)** | 17.9 | 2.1 | 8.6 | 5.7 | 10.7 | 3.6 | **8.1 (2.3)** | **7.2 (1.3)** | |
| *Tachyglossus aculeatus* | 0.0 | 0.0 | 0.0 | 0.0 | 0.0 | 0.0 | **0.0 (0.0)** | 2.9 | 0.0 | 3.6 | 0.7 | 0.7 | 0.7 | **1.4 (0.6)** | **0.7 (0.4)** | |
| *Perameles nasuta* | 0.7 | 2.9 | 1.4 | 1.4 | 2.1 | 1.4 | **1.7 (0.3)** | 5.7 | 4.3 | 4.3 | 0.7 | 3.6 | 0.7 | **3.2 (0.8)** | **2.4 (0.5)** | |
| *Thylogale sp*^2^*.* | 41.4 | 39.3 | 43.6 | 32.9 | 27.9 | 34.3 | **36.5 (2.4)** | 9.3 | 32.1 | 9.3 | 18.6 | 6.4 | 32.9 | **18.1 (4.9)** | **27.3 (3.8)** | |
| *Trichosurus sp.*^3^ | 10.7 | 6.4 | 10.0 | 17.9 | 16.4 | 10.0 | **11.9 (1.8)** | 26.4 | 10.0 | 33.6 | 35.0 | 27.1 | 30.0 | **27.0 (3.7)** | **19.5 (3.0)** | |
| *Varanus varius* | 0.7 | 0.0 | 0.7 | 0.0 | 0.0 | 0.0 | **0.2 (0.2)** | 0.0 | 0.7 | 0.0 | 0.0 | 0.0 | 0.2 | **0.1 (0.1)** | **0.2 (0.1)** | |

|  | Continuous forest | | | | | | | Fragments | | | | | | | | | All sites | | |
| --- | --- | --- | --- | --- | --- | --- | --- | --- | --- | --- | --- | --- | --- | --- | --- | --- | --- | --- | --- |
| Functional groups | 1 | 2 | 3 | 4 | 5 | 6 | **Av.** | | 1 | 2 | 3 | 4 | 5 | 6 | **Av.** | **Av.** | |  |  |
| Small birds | 4.3 | 5.3 | 5.7 | 7.9 | 5.0 | 5.3 | **6.6 (0.5)** | | 26.4 | 15.3 | 15.6 | 25.0 | 10.7 | 16.4 | **18.2 (2.5)** | **11.5 (2.4)** | | |  |
| Large birds | 12.9 | 5.7 | 10.0 | 12.1 | 14.3 | 14.3 | **11.5 (1.3)** | | 5.7 | 1.4 | 5.0 | 14.3 | 20.0 | 15.7 | **10.9 (3.0)** | **11.0 (1.7)** | |  |  |
| Small mammals | 22.9 | 35.7 | 28.6 | 45.7 | 41.4 | 39.3 | **35.6 (3.5)** | | 40.7 | 22.9 | 19.3 | 39.3 | 32.9 | 33.6 | **31.4 (3.5)** | **33.5 (3.7)** | |  |  |
| Large mammals | 10.7 | 6.4 | 10.0 | 17.9 | 16.4 | 10.0 | **11.9 (1.8)** | | 26.4 | 10.0 | 33.6 | 35.0 | 27.1 | 30.0 | **27.0 (3.7)** | **19.5 (3.3)** | |  |  |
| All birds | 16.4 | 10.0 | 15.0 | 18.6 | 19.3 | 17.1 | **16.1 (1.4)** | | 30.0 | 15.7 | 16.4 | 32.9 | 29.3 | 27.1 | **25.2 (3.0)** | **20.7 (2.7)** | |  |  |
| All mammals | 30.7 | 39.3 | 35.7 | 52.9 | 52.1 | 44.3 | **42.5 (3.6)** | | 57.9 | 29.3 | 47.9 | 62.1 | 50.7 | 52.9 | **50.1 (4.7)** | **46.3 (5.1)** | |  |  |
| All taxa | 42.9 | 43.6 | 45.0 | 62.1 | 60.0 | 53.6 | **51.2 (3.5)** | | 71.4 | 40.7 | 57.1 | 77.1 | 60.0 | 60.7 | **61.2 (5.2)** | **56.2 (6.1)** | |  |  |

^1^*M. cervinipes* is the only know *Melomys* species known to occur in the rainforests of the study region (Lott and Duggin 1993; Fine 2005)

^2^Most likely *T. thetis* however may potentially include *T. stigmatica*

^3^Most likely *T. caninus* or *T. vulpecula*

**S2 Table. Part B. Average percentage of days a given taxon was recorded by cameras with seeds (110 camera days/site).**

| Continuous forest Fragments All sites | | | | | | | | | | | | | | | |
| --- | --- | --- | --- | --- | --- | --- | --- | --- | --- | --- | --- | --- | --- | --- | --- |
| Vertebrate taxa | 1 | 2 | 3 | 4 | 5 | 6 | **Av.** | 1 | 2 | 3 | 4 | 5 | 6 | **Av.** | **Av.** |
| *Accipiter novaehollandiae* | 0.0 | 0.0 | 0.0 | 0.0 | 0.0 | 0.0 | **0.0 (0.0)** | 0.0 | 0.9 | 0.0 | 0.0 | 0.0 | 0.0 | **0.2 (0.2)** | **0.1 (0.1)** |
| *Chalcophaps indica* | 1.8 | 3.6 | 4.5 | 4.5 | 1.8 | 3.6 | **6.1 (0.5)** | 22.7 | 8.2 | 6.4 | 16.4 | 8.2 | 10.0 | **9.8 (2.6)** | **7.7 (1.8)** |
| *Columba leucomela* | 0.0 | 0.0 | 0.0 | 0.0 | 0.0 | 0.0 | **0.1 (0.0)** | 0.9 | 0.0 | 0.0 | 0.0 | 0.0 | 0.0 | **0.0 (0.2)** | **0.1 (0.1)** |
| *Eopsaltria australis* | 0.9 | 0.9 | 0.9 | 0.0 | 0.9 | 0.0 | **0.6 (0.2)** | 0.9 | 1.8 | 1.8 | 0.9 | 1.8 | 0.0 | **1.3 (0.3)** | **0.9 (0.2)** |
| *Leucosarcia melanoleuca* | 2.7 | 1.8 | 0.9 | 3.6 | 3.6 | 1.8 | **3.8 (0.4)** | 11.8 | 9.1 | 11.8 | 16.4 | 6.4 | 11.8 | **11.1 (1.4)** | **6.8 (1.5)** |
| *Orthonyx temminckii* | 3.6 | 1.8 | 0.0 | 1.8 | 0.9 | 0.9 | **1.3 (0.5)** | 0.0 | 0.0 | 0.9 | 0.9 | 6.4 | 0.0 | **1.6 (1.0)** | **1.4 (0.5)** |
| *Pitta versicolor* | 0.9 | 5.5 | 4.5 | 4.5 | 3.6 | 0.9 | **4.4 (0.8)** | 10.9 | 9.1 | 4.5 | 6.4 | 4.5 | 6.4 | **6.2 (1.0)** | **5.2 (0.8)** |
| *Psophodes olivaceus* | 1.8 | 1.8 | 0.0 | 0.9 | 2.7 | 0.0 | **1.6 (0.4)** | 3.6 | 1.8 | 2.7 | 0.9 | 5.5 | 0.9 | **2.4 (0.7)** | **1.9 (0.5)** |
| *Sericornis citreogularis* | 2.7 | 0.0 | 0.0 | 0.0 | 0.0 | 0.0 | **0.4 (0.5)** | 0.0 | 0.9 | 0.9 | 0.0 | 12.7 | 1.8 | **3.3 (2.0)** | **1.6 (1.0)** |
| *Sericulus chrysocephalus* | 0.0 | 0.0 | 0.0 | 0.0 | 0.0 | 0.0 | **0.1 (0.0)** | 0.9 | 0.0 | 0.9 | 0.0 | 0.0 | 0.0 | **0.2 (0.2)** | **0.2 (0.1)** |
| *Zoothera heinei* | 0.0 | 2.7 | 0.9 | 0.0 | 0.0 | 0.0 | **0.5 (0.4)** | 0.0 | 0.0 | 0.0 | 0.0 | 0.0 | 0.0 | **0.0 (0.0)** | **0.3 (0.2)** |
| *Alectura lathami* | 15.5 | 6.4 | 11.8 | 14.5 | 13.6 | 17.3 | **12.2 (1.6)** | 6.4 | 1.8 | 5.5 | 15.5 | 21.8 | 18.2 | **12.5 (3.3)** | **12.3 (1.7)** |
| *Antechinus sp.* | 0.0 | 6.4 | 2.7 | 1.8 | 0.0 | 0.9 | **1.7 (1.0)** | 0.0 | 0.0 | 0.0 | 0.0 | 0.0 | 0.0 | **0.0 (0.0)** | **1.0 (0.6)** |
| *Melomys cervinipes*^1^ | 2.7 | 5.5 | 0.9 | 0.9 | 2.7 | 6.4 | **2.7 (0.9)** | 0.0 | 0.0 | 0.0 | 0.0 | 0.0 | 0.0 | **0.0 (0.0)** | **1.6 (0.7)** |
| *Rattus fuscipes* | 20.0 | 33.6 | 24.5 | 46.4 | 38.2 | 40.9 | **33.5 (4.1)** | 30.9 | 2.7 | 10.0 | 16.4 | 20.9 | 12.7 | **12.5 (4.0)** | **24.8 (3.9)** |
| *Rattus rattus* | 6.4 | 6.4 | 0.9 | 4.5 | 0.0 | 0.0 | **4.7 (1.3)** | 14.5 | 21.8 | 9.1 | 30.0 | 14.5 | 30.0 | **21.1 (3.6)** | **11.5 (3.1)** |
| *Rattus sp.* | 0.9 | 8.2 | 9.1 | 11.8 | 9.1 | 7.3 | **9.2 (1.5)** | 18.2 | 1.8 | 10.0 | 7.3 | 13.6 | 4.5 | **7.5 (2.5)** | **8.5 (1.4)** |
| *Tachyglossus aculeatus* | 0.0 | 0.0 | 0.0 | 0.0 | 0.0 | 0.0 | **0.3 (0.0)** | 1.8 | 0.0 | 4.5 | 0.9 | 0.9 | 0.9 | **1.5 (0.6)** | **0.8 (0.4)** |
| *Perameles nasuta* | 0.9 | 3.6 | 1.8 | 1.8 | 2.7 | 1.8 | **2.9 (0.4)** | 7.3 | 4.5 | 5.5 | 0.9 | 4.5 | 0.9 | **3.3 (1.0)** | **3.0 (0.6)** |
| *Thylogale sp.*^2^ | 27.3 | 31.8 | 35.5 | 28.2 | 23.6 | 23.6 | **25.3 (1.9)** | 7.3 | 15.5 | 10.0 | 17.3 | 5.5 | 21.8 | **14.0 (2.6)** | **20.6 (2.8)** |
| *Trichosurus sp.*^3^ | 10.0 | 4.5 | 9.1 | 19.1 | 12.7 | 10.9 | **13.0 (2.0)** | 24.5 | 10.9 | 33.6 | 37.3 | 31.8 | 31.8 | **29.1 (3.9)** | **19.7 (3.3)** |
| *Varanus varius* | 0.9 | 0.0 | 0.9 | 0.0 | 0.0 | 0.0 | **0.3 (0.2)** | 0.0 | 0.0 | 0.9 | 0.0 | 0.0 | 0.0 | **0.2 (0.2)** | **0.2 (0.1)** |

^1^*M. cervinipes* is the only know *Melomys* species known to occur in the rainforests of the study region (Lott and Duggin 1993; Fine 2005)

^2^Most likely *T. thetis* however may potentially include *T. stigmatica*

^3^Most likely *T. caninus* or *T. vulpecula*

**S2 Table. Part C. Average percentage of days a given taxon was recorded by cameras without seeds (30 camera days/site).**

|  | Continuous forest | | | | | | Fragments | | | | | | | | All sites | |
| --- | --- | --- | --- | --- | --- | --- | --- | --- | --- | --- | --- | --- | --- | --- | --- | --- |
| Vertebrate taxa | 1 | 2 | 3 | 4 | 5 | 6 | **Av.** | 1 | 2 | 3 | 4 | 5 | 6 | **Av.** | **Av.** |  |
| *Accipiter novaehollandiae* | 0.0 | 0.0 | 0.0 | 0.0 | 0.0 | 0.0 | **0.0 (0.0)** | 0.0 | 0.0 | 0.0 | 0.0 | 0.0 | 0.0 | **0.0 (0.0)** | **0.0 (0.0)** |  |
| *Chalcophaps indica* | 0.0 | 0.0 | 0.0 | 3.3 | 0.0 | 0.0 | **0.6 (0.5)** | 6.7 | 3.3 | 0.0 | 3.3 | 0.0 | 6.7 | **3.3 (1.2)** | **1.9 (0.8)** |  |
| *Columba leucomela* | 0.0 | 0.0 | 0.0 | 0.0 | 0.0 | 0.0 | **0.0 (0.0)** | 0.0 | 0.0 | 0.0 | 0.0 | 0.0 | 0.0 | **0.0 (0.0)** | **0.0 (0.0)** |  |
| *Eopsaltria australis* | 0.0 | 0.0 | 0.0 | 0.0 | 0.0 | 0.0 | **0.0 (0.0)** | 0.0 | 0.0 | 0.0 | 3.3 | 0.0 | 0.0 | **0.6 (0.6)** | **0.3 (0.3)** |  |
| *Leucosarcia melanoleuca* | 3.3 | 0.0 | 6.7 | 3.3 | 3.3 | 0.0 | **2.8 (0.9)** | 0.0 | 0.0 | 0.0 | 0.0 | 0.0 | 0.0 | **0.0 (0.0)** | **1.4 (0.6)** |  |
| *Orthonyx temminckii* | 0.0 | 0.0 | 0.0 | 0.0 | 0.0 | 0.0 | **0.0 (0.0)** | 0.0 | 0.0 | 0.0 | 0.0 | 0.0 | 0.0 | **0.0 (0.0)** | **0.0 (0.0)** |  |
| *Pitta versicolor* | 0.0 | 0.0 | 0.0 | 0.0 | 0.0 | 0.0 | **0.0 (0.0)** | 0.0 | 3.3 | 0.0 | 3.3 | 0.0 | 0.0 | **1.1 (0.7)** | **0.6 (0.4)** |  |
| *Psophodes olivaceus* | 0.0 | 0.0 | 0.0 | 6.7 | 0.0 | 3.3 | **1.7 (1.0)** | 0.0 | 0.0 | 0.0 | 0.0 | 0.0 | 6.7 | **1.1 (1.1)** | **1.4 (0.8)** |  |
| *Sericornis citreogularis* | 0.0 | 0.0 | 0.0 | 3.3 | 0.0 | 0.0 | **0.6 (0.5)** | 0.0 | 0.0 | 0.0 | 0.0 | 0.0 | 6.7 | **1.1 (1.1)** | **0.8 (0.6)** |  |
| *Sericulus chrysocephalus* | 0.0 | 0.0 | 0.0 | 0.0 | 0.0 | 0.0 | **0.0 (0.0)** | 0.0 | 0.0 | 0.0 | 0.0 | 0.0 | 0.0 | **0.0 (0.0)** | **0.0 (0.0)** |  |
| *Zoothera heinei* | 0.0 | 0.0 | 0.0 | 0.0 | 0.0 | 0.0 | **0.0 (0.0)** | 0.0 | 0.0 | 0.0 | 0.0 | 0.0 | 0.0 | **0.0 (0.0)** | **0.0 (0.0)** |  |
| *Alectura lathami* | 3.3 | 3.3 | 3.3 | 3.3 | 16.7 | 3.3 | **5.6 (2.0)** | 3.3 | 0.0 | 3.3 | 10.0 | 13.3 | 6.7 | **6.1 (2.0)** | **5.8 (1.4)** |  |
| *Antechinus sp.* | 0.0 | 0.0 | 0.0 | 0.0 | 0.0 | 0.0 | **0.0 (0.0)** | 0.0 | 0.0 | 0.0 | 0.0 | 0.0 | 0.0 | **0.0 (0.0)** | **0.0 (0.0)** |  |
| *Melomys cervinipes*^1^ | 0.0 | 0.0 | 0.0 | 0.0 | 0.0 | 0.0 | **0.0 (0.0)** | 0.0 | 0.0 | 0.0 | 0.0 | 0.0 | 0.0 | **0.0 (0.0)** | **0.0 (0.0)** |  |
| *Rattus fuscipes* | 6.7 | 10.0 | 16.7 | 13.3 | 20.0 | 3.3 | **11.7 (2.3)** | 0.0 | 0.0 | 0.0 | 0.0 | 0.0 | 0.0 | **0.0 (0.0)** | **5.8 (2.1)** |  |
| *Rattus rattus* | 0.0 | 0.0 | 0.0 | 0.0 | 0.0 | 0.0 | **0.0 (0.0)** | 10.0 | 10.0 | 0.0 | 13.3 | 6.7 | 13.3 | **8.9 (2.0)** | **4.4 (1.7)** |  |
| *Rattus sp.* | 0.0 | 0.0 | 3.3 | 0.0 | 3.3 | 0.0 | **1.1 (0.6)** | 16.7 | 3.3 | 3.3 | 0.0 | 0.0 | 0.0 | **3.9 (2.6)** | **2.5 (1.4)** |  |
| *Tachyglossus aculeatus* | 0.0 | 0.0 | 0.0 | 0.0 | 0.0 | 0.0 | **0.0 (0.0)** | 6.7 | 0.0 | 0.0 | 0.0 | 0.0 | 0.0 | **1.1 (1.1)** | **0.6 (0.6)** |  |
| *Perameles nasuta* | 0.0 | 0.0 | 0.0 | 0.0 | 0.0 | 0.0 | **0.0 (0.0)** | 0.0 | 3.3 | 0.0 | 0.0 | 0.0 | 0.0 | **0.6 (0.4)** | **0.3 (0.3)** |  |
| *Thylogale sp.*^2^ | 46.7 | 36.7 | 56.7 | 40.0 | 23.3 | 43.3 | **41.1 (4.1)** | 10.0 | 56.7 | 6.7 | 23.3 | 6.7 | 16.7 | **20.0 (7.8)** | **30.6 (5.3)** |  |
| *Trichosurus sp.*^3^ | 13.3 | 13.3 | 13.3 | 13.3 | 30.0 | 6.7 | **15.0 (2.9)** | 33.3 | 6.7 | 33.3 | 26.7 | 10.0 | 23.3 | **22.2 (4.7)** | **18.6 (2.9)** |  |
| *Varanus varius* | 0.0 | 0.0 | 0.0 | 0.0 | 0.0 | 0.0 | **0.0 (0.0)** | 0.0 | 0.0 | 0.0 | 0.0 | 0.0 | 0.0 | **0.0 (0.0)** | **0.0 (0.0)** |  |

^1^*M. cervinipes* is the only know *Melomys* species known to occur in the rainforests of the study region (Lott and Duggin 1993; Fine 2005)

^2^Most likely *T. thetis* however may potentially include *T. stigmatica*

^3^Most likely *T. caninus* or *T. vulpecula*

**S2 Table. Part D. Non-destructive interest scores of vertebrate taxa and predator functional groups.**

|  | Continuous forest | | | | | | | Fragments | | | | | | | All sites |
| --- | --- | --- | --- | --- | --- | --- | --- | --- | --- | --- | --- | --- | --- | --- | --- |
| Vertebrate taxa | 1 | 2 | 3 | 4 | 5 | 6 | **Av.** | 1 | 2 | 3 | 4 | 5 | 6 | **Av.** | **Av.** |
| *Accipiter novaehollandiae* | N | N | N | N | N | N | **N** | 0.00 | N | N | N | 0.00 | 0.00 | **0 (0.00)** | **0 (0.00)** |
| *Chalcophaps indica* | 0.00 | 0.00 | 0.00 | 0.00 | 0.00 | 0.00 | **0.00 (0.00)** | 0.00 | 0.16 | 0.11 | 0.29 | 0.00 | 0.00 | **0.09 (0.05)** | **0.05 (0.03)** |
| *Columba leucomela* | N | N | N | N | N | N | **N** | 0.00 | N | N | N | N | 0.00 | **0.00 (0.00)** | **0.00 (0.00)** |
| *Eopsaltria australis* | 0.00 | 0.00 | 0.00 | N | 0.00 | N | **0.00** | N | 0.00 | 0.00 | 0.00 | 0.00 | 0.00 | **0.00 (0.00)** | **0.00 (0.00)** |
| *Leucosarcia melanoleuca* | 0.00 | 0.00 | 0.00 | 0.00 | 0.00 | 0.00 | **0.00** | 0.00 | 0.15 | 0.00 | 0.15 | 0.00 | 0.14 | **0.08 (0.03)** | **0.04 (0.02)** |
| *Orthonyx temminckii* | 0.00 | 0.00 | N | 0.00 | 0.00 | 0.00 | **0.00** | N | N | N | 0.00 | 0.00 | 0.00 | **0.00 (0.00)** | **0.00 (0.00)** |
| *Pitta versicolor* | 0.00 | 0.00 | 0.00 | 0.00 | 0.00 | 0.00 | **0.00** | 0.00 | 0.00 | 0.00 | 0.20 | 0.00 | 0.00 | **0.03 (0.03)** | **0.02 (0.02)** |
| *Psophodes olivaceus* | 0.50 | 0.00 | N | 0.00 | 0.00 | N | **0.13 (0.10)** | 0.00 | 0.00 | 0.00 | 0.00 | 0.00 | 0.00 | **0.00 (0.00)** | **0.05 (0.05)** |
| *Sericornis citreogularis* | 0.00 | N | N | N | N | N | **0.00** | 0.00 | N | 0.00 | 0.00 | N | 0.00 | **0.00 (0.00)** | **0.00 (0.00)** |
| *Sericulus chrysocephalus* | N | N | N | N | N | N | **N** | 0.00 | N | 0.00 | N | N | 0.00 | **0.00 (0.00)** | **0.00 (0.00)** |
| *Zoothera heinei* | 0.00 | 0.00 | N | N | N | 0.00 | **0.00** | N | N | N | N | N | N | **N** | **N** |
| *Alectura lathami* | 0.00 | 0.00 | 0.00 | 0.13 | 0.00 | 0.05 | **0.03 (0.02)** | 0.10 | 0.00 | 0.00 | 0.00 | 0.06 | 0.04 | **0.03 (0.02)** | **0.03 (0.01)** |
| *Antechinus sp.* | N | 0.00 | 0.00 | 0.00 | N | 0.00 | **0.00 (0.00)** | N | N | N | N | N | N | **N** | **0.00 (0.00)** |
| *Melomys cervinipes*^1^ | 0.00 | 0.00 | 0.00 | 0.00 | 0.33 | 0.29 | **0.10 (0.07)** | N | N | N | N | N | N | **N** | **0.10 (0.05)** |
| *Rattus fuscipes* | 0.36 | 0.27 | 0.59 | 0.24 | 0.67 | 0.18 | **0.38 (0.08)** | 0.29 | 0.35 | 0.33 | 0.45 | 0.06 | 0.39 | **0.31 (0.06)** | **0.35 (0.05)** |
| *Rattus rattus* | 0.29 | 0.29 | 0.00 | 0.20 | N | N | **0.19 (0.06)** | 0.18 | 0.25 | 0.21 | 0.00 | 0.12 | 0.31 | **0.18 (0.04)** | **0.18 (0.03)** |
| *Rattus sp.* | 0.00 | 0.33 | 0.20 | 0.15 | 0.20 | 0.00 | **0.15 (0.05)** | 0.00 | 0.35 | 0.00 | 0.18 | 0.25 | 0.00 | **0.13 (0.06)** | **0.14 (0.04)** |
| *Tachyglossus aculeatus* | N | N | N | N | N | N | **N** | 0.00 | N | 0.00 | 0.00 | 0.00 | 0.00 | **0.00 (0.00)** | **0.00 (0.00)** |
| *Perameles nasuta* | 0.00 | 0.25 | 0.00 | 0.00 | 0.33 | 0.00 | **0.10 (0.06)** | 0.00 | 0.13 | 0.00 | 0.00 | 0.00 | 0.00 | **0.02 (0.02)** | **0.06 (0.03)** |
| *Thylogale sp.*^2^ | 0.33 | 0.40 | 0.36 | 0.32 | 0.12 | 0.23 | **0.29 (0.04)** | 0.29 | 0.38 | 0.41 | 0.18 | 0.11 | 0.50 | **0.31 (0.06)** | **0.30 (0.04)** |
| *Trichosurus sp.*^3^ | 0.27 | 0.20 | 0.20 | 0.19 | 0.21 | 0.42 | **0.25 (0.04)** | 0.26 | 0.44 | 0.17 | 0.30 | 0.29 | 0.57 | **0.34 (0.06)** | **0.29 (0.04)** |
| *Varanus varius* | 0.00 | N | 0.00 | N | N | N | **0.00 (0.00)** | N | N | N | 0.00 | N | N | **0.00 (0.00)** | **0.00 (0.00)** |

|  | Continuous forest | | | | | | Fragments All sites | | | | | | | | |  |
| --- | --- | --- | --- | --- | --- | --- | --- | --- | --- | --- | --- | --- | --- | --- | --- | --- |
| Seed predator functional  groups | Big Scrub 1 | Big Scrub  2 | Big Scrub  3 | Big Scrub  4 | Boome-rang Falls | Minyon Falls | **Cont**  **av.** | Booyong Flora Reserve | Davis Scrub | Dawes Bush | Emery's Scrub | Johnson's Nature Reserve | Victoria Park | **Frag**  **av.** | **Overall**  **av.** |  |
| Small birds | 0.00 | 0.00 | 0.00 | 0.00 | 0.00 | 0.00 | **0.00 (0.00)** | 0.17 | 0.05 | 0.21 | 0.00 | 0.06 | 0.00 | **0.08 (0.04)** | **0.04 (0.02)** |  |
| Large birds | 0.00 | 0.00 | 0.00 | 0.13 | 0.00 | 0.05 | **0.03 (0.02)** | 0.00 | 0.00 | 0.00 | 0.06 | 0.04 | 0.10 | **0.03 (0.02)** | **0.03 (0.01)** |  |
| Small mammals | 0.33 | 0.28 | 0.56 | 0.23 | 0.61 | 0.19 | **0.37 (0.07)** | 0.36 | 0.21 | 0.27 | 0.14 | 0.32 | 0.22 | **0.25 (0.03)** | **0.31 (0.04)** |  |
| Large mammals | 0.27 | 0.20 | 0.20 | 0.19 | 0.21 | 0.42 | **0.25 (0.04)** | 0.44 | 0.17 | 0.30 | 0.29 | 0.29 | 0.26 | **0.29 (0.04)** | **0.27 (0.03)** |  |
| All birds | 0.00 | 0.00 | 0.00 | 0.09 | 0.00 | 0.04 | **0.02 (0.01)** | 0.15 | 0.05 | 0.18 | 0.02 | 0.06 | 0.06 | **0.09 (0.03)** | **0.05 (0.02)** |  |
| All mammals | 0.35 | 0.29 | 0.53 | 0.26 | 0.59 | 0.24 | **0.38 (0.06)** | 0.41 | 0.22 | 0.32 | 0.26 | 0.33 | 0.28 | **0.30 (0.03)** | **0.34 (0.03)** |  |

^1^*M. cervinipes* is the only know *Melomys* species known to occur in the rainforests of the study region (Lott and Duggin 1993; Fine 2005)

^2^Most likely *T. thetis* however may potentially include *T. stigmatica*

^3^Most likely *T. caninus* or *T. vulpecula*

**S2 Table. Part E. Destructive interest scores of vertebrate taxa and predator functional groups.**

| Continuous forest | | | | | | | | Fragments | | | | | | | **All sites** |
| --- | --- | --- | --- | --- | --- | --- | --- | --- | --- | --- | --- | --- | --- | --- | --- |
| Vertebrate taxa | 1 | 2 | 3 | 4 | 5 | 6 | **Av.** | 1 | 2 | 3 | 4 | 5 | 6 | **Av.** | **Av.** |
| *Accipiter novaehollandiae* | N | N | N | N | N | N | **N** | N | 0.00 | N | N | N | N | **0.00 (0.04)** | **0.00 (0.00)** |
| *Chalcophaps indica* | 0.00 | 0.00 | 0.20 | 0.00 | 0.00 | 0.50 | **0.12 (0.08)** | 0.16 | 0.33 | 0.43 | 0.22 | 0.22 | 0.27 | **0.27 (0.00)** | **0.19 (0.05)** |
| *Columba leucomela* | N | N | N | N | N | N | **0.00 (0.00)** | 0.00 | N | N | N | N | N | **0.00 (0.00)** | **0.00 (0.00)** |
| *Eopsaltria australis* | 0.00 | 0.00 | 0.00 | N | 0.00 | N | **0.00 (0.00)** | 0.00 | 0.00 | 0.00 | 0.00 | 0.00 | N | **0.00 (0.04)** | **0.00 (0.00)** |
| *Leucosarcia melanoleuca* | 0.00 | 0.00 | 0.00 | 0.00 | 0.25 | 0.00 | **0.04 (0.04)** | 0.31 | 0.30 | 0.38 | 0.50 | 0.29 | 0.23 | **0.33 (0.00)** | **0.19 (0.05)** |
| *Orthonyx temminckii* | 0.00 | 0.00 | N | 0.00 | 0.00 | 0.00 | **0.00 (0.00)** | N | N | 0.00 | 0.00 | 0.00 | N | **0.00 (0.00)** | **0.00 (0.00)** |
| *Pitta versicolor* | 0.00 | 0.00 | 0.00 | 0.00 | 0.00 | 0.00 | **0.00 (0.00)** | 0.00 | 0.00 | 0.00 | 0.00 | 0.00 | 0.00 | **0.00 (0.00)** | **0.00 (0.00)** |
| *Psophodes olivaceus* | 0.00 | 0.00 | N | 0.00 | 0.00 | N | **0.00 (0.00)** | 0.00 | 0.00 | 0.00 | 0.00 | 0.00 | 0.00 | **0.00 (0.00)** | **0.00 (0.00)** |
| *Sericornis citreogularis* | 0.00 | N | N | N | N | N | **0.00 (0.00)** | N | 0.00 | 0.00 | N | 0.00 | 0.00 | **0.00 (0.00)** | **0.00 (0.00)** |
| *Sericulus chrysocephalus* | N | N | N | N | N | N | **N** | 0.00 | N | 0.00 | N | N | N | **0.00 (0.00)** | **0.00 (0.00)** |
| *Zoothera heinei* | N | 0.00 | 0.00 | N | N | N | **0.00 (0.00)** | N | N | N | N | N | N | **0.00 (0.00)** | **0.00 (0.00)** |
| *Alectura lathami* | 0.47 | 0.14 | 0.62 | 0.25 | 0.53 | 0.37 | **0.40 (0.07)** | 0.57 | 0.50 | 0.00 | 0.41 | 0.42 | 0.30 | **0.37 (0.08)** | **0.38 (0.05)** |
| *Antechinus sp.* | N | 0.00 | 0.00 | 0.00 | N | 0.00 | **0.00 (0.00)** | N | N | N | N | N | N | **N** | **0.00 (0.00)** |
| *Melomys cervinipes*^1^ | 0.25 | 0.33 | 0.37 | 0.43 | 0.33 | 0.10 | **0.30 (0.04)** | N | N | N | N | N | N | **N** | **0.30 (0.03)** |
| *Rattus fuscipes* | 0.59 | 0.32 | 0.56 | 0.57 | 0.50 | 0.47 | **0.50 (0.04)** | 0.41 | 0.17 | 0.36 | 0.39 | 0.22 | 0.50 | **0.34 (0.05)** | **0.42 (0.04)** |
| *Rattus rattus* | 0.29 | 0.29 | 0.00 | 0.00 | N | N | **0.14 (0.06)** | 0.25 | 0.42 | 0.30 | 0.35 | 0.38 | 0.24 | **0.32 (0.03)** | **0.25 (0.04)** |
| *Rattus sp.* | 0.00 | 0.11 | 0.20 | 0.00 | 0.10 | 0.00 | **0.07 (0.03)** | 0.20 | 0.00 | 0.27 | 0.25 | 0.27 | 0.20 | **0.20 (0.04)** | **0.13 (0.03)** |
| *Tachyglossus aculeatus* | N | N | N | N | N | N | **N** | 0.00 | N | 0.00 | 0.00 | 0.00 | 0.00 | **0.00 (0.00)** | **0.00 (0.00)** |
| *Perameles nasuta* | 0.00 | 0.00 | 0.00 | 0.00 | 0.00 | 0.00 | **0.00 (0.00)** | 0.00 | 0.00 | 0.00 | 0.00 | 0.00 | 0.00 | **0.00 (0.00)** | **0.00 (0.00)** |
| *Thylogale sp.*^2^ | 0.00 | 0.03 | 0.03 | 0.03 | 0.00 | 0.00 | **0.01 (0.01)** | 0.00 | 0.00 | 0.09 | 0.00 | 0.00 | 0.00 | **0.02 (0.02)** | **0.01 (0.01)** |
| *Trichosurus sp.*^3^ | 0.27 | 0.20 | 0.30 | 0.10 | 0.21 | 0.25 | **0.22 (0.03)** | 0.22 | 0.25 | 0.41 | 0.27 | 0.17 | 0.43 | **0.29 (0.04)** | **0.26 (0.03)** |
| *Varanus varius* | 0.00 | N | 0.00 | N | N | N | **0.00 (0.00)** | N | N | 0.00 | N | N | N | **0.00 (0.00)** | **0.00 (0.00)** |

|  | Continuous forest | | | | | | Fragments | | | | | | | | **All sites** |  |
| --- | --- | --- | --- | --- | --- | --- | --- | --- | --- | --- | --- | --- | --- | --- | --- | --- |
| Seed predator functional groups | Big Scrub 1 | Big Scrub 2 | Big Scrub 3 | Big Scrub  4 | Boome-rang Falls | Minyon Falls | **Cont**  **av.** | Booyong  Flora Reserve | Davis Scrub | Dawes Bush | Emery's Scrub | Johnson's Nature Reserve | Victoria Park | **Frag**  **av.** | **Overall**  **av.** | |
| Large birds | 0.47 | 0.14 | 0.62 | 0.25 | 0.53 | 0.37 | **0.40 (0.07)** | 0.57 | 0.50 | 0.00 | 0.41 | 0.42 | 0.30 | **0.37 (0.08)** | **0.38 (0.05)** | |
| Small birds | 0.00 | 0.00 | 0.17 | 0.00 | 0.17 | 0.33 | **0.11 (0.05)** | 0.23 | 0.32 | 0.42 | 0.38 | 0.25 | 0.26 | **0.31 (0.03)** | **0.21 (0.04)** | |
| Small mammals | 0.50 | 0.35 | 0.53 | 0.50 | 0.43 | 0.41 | **0.45 (0.03)** | 0.40 | 0.38 | 0.36 | 0.41 | 0.33 | 0.35 | **0.37 (0.01)** | **0.41 (0.02)** | |
| Large mammals | 0.27 | 0.20 | 0.30 | 0.10 | 0.21 | 0.25 | **0.22 (0.03)** | 0.22 | 0.25 | 0.41 | 0.27 | 0.17 | 0.43 | **0.29 (0.04)** | **0.26 (0.03)** | |
| All birds | 0.38 | 0.08 | 0.44 | 0.17 | 0.43 | 0.39 | **0.32 (0.06)** | 0.31 | 0.33 | 0.36 | 0.43 | 0.35 | 0.31 | **0.35 (0.02)** | **0.33 (0.03)** | |
| All mammals | 0.46 | 0.35 | 0.50 | 0.48 | 0.43 | 0.42 | **0.44 (0.02)** | 0.36 | 0.38 | 0.42 | 0.41 | 0.27 | 0.43 | **0.38 (0.02)** | **0.41 (0.02)** | |
| All predators | 0.45 | 0.31 | 0.51 | 0.45 | 0.49 | 0.46 | **0.45 (0.03)** | 0.40 | 0.39 | 0.43 | 0.52 | 0.38 | 0.43 | **0.42 (0.02)** | **0.44 (0.02)** | |

^1^*M. cervinipes* is the only know *Melomys* species known to occur in the rainforests of the study region (Lott and Duggin 1993; Fine 2005)

^2^Most likely *T. thetis* however may potentially include *T. stigmatica*

^3^Most likely *T. caninus* or *T. vulpecula*

**S2 Table. Part F.** **Proportion of seeds removed or damaged after five days of exposure at seed stations, for each seed species.**

| Seed species | Seed size category | Av. prop. removed or damaged (SE) |
| --- | --- | --- |
| *Melia azedarach* | S | 0.58 (0.05) |
| *Denhamia celastroides* | S | 0.44 (0.06) |
| *Tabernaemontana pandacaqui* | S | 0.61 (0.06) |
| *Eupomatia laurina* | S | 0.46 (0.04) |
| *Guioa semiglauca* | S | 0.43 (0.07) |
| *Mallotus philippensis* | S | 0.76 (0.06) |
| *Atractocarpus chartaceus* | S | 0.51 (0.05) |
| *Ehretia acuminate** | S | 0.23 (0.03) |
| *Bachychiton acerifolius* | S | 0.60 (0.07) |
| *Cinnamomum camphora* | S | 0.68 (0.04) |
| *Neolitsea dealbata* | S | 0.62 (0.05) |
| *Cryptocarya microneura* | L | 0.53 (0.06) |
| *Wilkea huegeliana* | L | 0.39 (0.05) |
| *Cryptocarya obovata* | L | 0.52 (0.04) |
| *Acmena hemilampra* | L | 0.46 (0.05) |
| *Cryptocarya glaucescens** | L | 0.56 (0.03) |
| *Podocarpus elatus* | L | 0.69 (0.07) |
| *Castanospora alphandii* | L | 0.44 (0.05) |
| *Acmena ingens* | L | 0.33 (0.04) |
| *Castanospermum australe* | L | 0.74 (0.08) |

**S2 Table. Part G.** **Proportion of seeds removed or damaged after five days of exposure at seed stations, for each site.**

|  | | Continuous forest | | | | | | | | Fragments | | | | | | | | **Overall** |  |  |
| --- | --- | --- | --- | --- | --- | --- | --- | --- | --- | --- | --- | --- | --- | --- | --- | --- | --- | --- | --- | --- |
|  | | 1 | | 2 | 3 | 4 | 5 | 6 | **Av.** |  | | 1 | 2 | 3 | 4 | 5 | 6 | **Av.** | | |
| Small seeds (11 species) | | 0.52 | | 0.50 | 0.50 | 0.54 | 0.51 | 0.49 | **0.51 (0.01)** | 0.56 | | 0.47 | 0.46 | 0.53 | 0.52 | 0.55 | **0.51 (0.01)** | **0.51 (0.01)** | | |
| Large seeds (9 species) | | 0.54 | | 0.48 | 0.53 | 0.38 | 0.54 | 0.42 | **0.48 (0.02)** | 0.76 | | 0.48 | 0.48 | 0.56 | 0.60 | 0.51 | **0.56 (0.04)** | **0.52 (0.02)** | | |
| All seeds (20 species) | | 0.53 | | 0.49 | 0.52 | 0.47 | 0.53 | 0.45 | **0.50 (0.01)** | 0.65 | | 0.47 | 0.47 | 0.54 | 0.56 | 0.53 | **0.54 (0.02)** | **0.52 (0.01)** | | |

**S2 Table. Part H. Sampling rates of small insectivorous birds.**

|  | Continuous forest | | | | | | | Fragments | | | | | | |  | |
| --- | --- | --- | --- | --- | --- | --- | --- | --- | --- | --- | --- | --- | --- | --- | --- | --- |
| Species | 1 | 2 | 3 | 4 | 5 | 6 | **Av.** | 1 | 2 | 3 | 4 | 5 | 6 | **Av.** | ***t*^1^** | ***P*** |
| *Eopsaltria australis* | 0.71 | 0.71 | 0.71 | 0.00 | 0.71 | 0.00 | **0.48 (0.14)** | 0.71 | 1.43 | 1.43 | 1.43 | 1.43 | 0.00 | **1.07 (0.24)** | -2.13 | 0.03 |
| *Orthonyx temminckii* | 2.86 | 1.43 | 0.00 | 1.43 | 0.71 | 0.71 | **1.19 (0.36)** | 0.00 | 0.00 | 0.71 | 0.71 | 5.00 | 0.00 | **1.07 (0.79)** | -0.44 | 0.34 |
| *Pitta versicolor* | 0.71 | 4.29 | 3.57 | 3.57 | 2.86 | 0.71 | **2.62 (0.58)** | 8.57 | 8.57 | 3.57 | 5.71 | 3.57 | 5.00 | **5.83 (0.92)** | -2.07 | 0.03 |
| *Psophodes olivaceus* | 1.43 | 1.43 | 0.00 | 2.14 | 2.14 | 0.71 | **1.31 (0.31)** | 2.86 | 1.43 | 2.14 | 0.71 | 4.29 | 2.14 | **2.26 (0.50)** | -1.17 | 0.14 |
| *Sericornis citreogularis* | 2.14 | 0.00 | 0.00 | 0.71 | 0.00 | 0.00 | **0.48 (0.32)** | 0.00 | 0.71 | 0.71 | 0.00 | 10.00 | 2.86 | **2.38 (1.58)** | -1.46 | 0.09 |
| All species | 7.14 | 10.00 | 5.00 | 7.85 | 6.42 | 2.14 | **6.42 (1.00)** | 12.14 | 11.42 | 8.57 | 7.85 | 17.85 | 9.28 | **11.19 (1.49)** | -2.09 | 0.03 |

From: Palmer GJ. Interactions between rainforest trees and their vertebrate seed predators in continuous and fragmented habitat. PhD thesis. Griffith University, Brisbane. 2016 (unpublished).

^1^Results from independent-sample t-tests. *P* values are bolded if statistically significant (*P* < 0.05).
