# Supplementary material for: Impacts of rainforest fragmentation on the composition of ground-active vertebrate communities and their patterns of seed consumption
Source: PLoS One. 2018 Sep 12;13(9):e0202870. doi: 10.1371/journal.pone.0202870 (PMC6135387; doi:10.1371/journal.pone.0202870)
Supplement: S3 Table — Rainforest patch size is the area of rainforest habitat, which is surrounded by pasture in the case of fragments and by drier eucalypt forest in the case of continuous forest. (DOCX) [file pone.0202870.s003.docx]

**S3 Table. Effect of rainforest patch size on sampling rates and feeding behaviours of seed predator species and functional groups.**

| **Type of seed predator** | **Sampling rate^1^** | | | | **Destructive interest^2^** | | | |
| --- | --- | --- | --- | --- | --- | --- | --- | --- |
|  | **simple^3^** | | **log^4^** | | **simple^3^** | | **log^4^** | |
|  | ***r*** | ***P*** | ***r*** | ***P*** | ***r*** | ***P*** | ***r*** | ***P*** |
| Individual species: |  |  |  |  |  |  |  |  |
| *Alectura lathami* | 0.03 | 0.92 | 0.23 | 0.47 | 0.03 | 0.92 | 0.25 | 0.43 |
| *Chalcophaps indica* | 0.64 | **0.03** | 0.78 | **0.003** | 0.58 | **0.05** | 0.67 | **0.02** |
| *Leucosarcia melanoleuca* | 0.83 | **0.001** | 0.91 | **0.0001** | 0.89 | **0.0001** | 0.89 | **0.0001** |
| *Melomys cervinipes* | 0.56 | 0.06 | 0.47 | 0.12 | 0.53 | 0.07 | 0.56 | 0.06 |
| *Rattus fuscipes* | 0.64 | **0.03** | 0.65 | **0.02** | 0.56 | 0.06 | 0.43 | 0.16 |
| *Rattus* | 0.71 | **0.01** | 0.78 | **0.003** | 0.58 | **0.05** | 0.63 | **0.03** |
| *Trichosurus* sp. | 0.70 | **0.01** | 0.76 | **0.004** | 0.41 | 0.18 | 0.48 | 0.11 |
| Functional groups: |  |  |  |  |  |  |  |  |
| Small birds | 0.76 | **0.004** | 0.90 | **0.0001** | 0.84 | **0.0006** | 0.35 | 0.26 |
| Large birds | 0.03 | 0.92 | 0.23 | 0.47 | 0.03 | 0.92 | 0.25 | 0.43 |
| Small mammals | 0.13 | 0.68 | 0.19 | 0.55 | 0.67 | **0.02** | 0.57 | **0.05** |
| Large mammals | 0.70 | **0.01** | 0.76 | **0.004** | 0.41 | 0.18 | 0.48 | 0.11 |
| All birds | 0.67 | **0.02** | 0.64 | **0.03** | 0.32 | 0.31 | 0.34 | 0.28 |
| All mammals | 0.47 | 0.12 | 0.42 | 0.17 | 0.49 | 0.11 | 0.32 | 0.31 |
| All predators | 0.63 | **0.04** | 0.68 | **0.03** | 0.05 | 0.87 | 0.05 | 0.88 |

**Table legend and footnotes**

^1^Sampling rate (‘% days recorded’) is the percent of camera days a taxon was recorded by cameras at a given site (N=12 sites)

^2^Destructive interest is the percent of camera days in which a given taxon was recorded behaviourally interfering with seeds, calculated from the total number of days in which it was recorded at a given site (N=12 sites)

^3^From Pearson’s correlation analyses with sites as replicates (df=10) using untransformed data. *P* values are bolded if statistically significant (*P* < 0.05).

^4^From Pearson’s correlation analyses with sites as replicates (df=10) using log10 transformed data. *P* values are bolded if statistically significant (*P* < 0.05).
