# Supplementary material for: Impacts of rainforest fragmentation on the composition of ground-active vertebrate communities and their patterns of seed consumption
Source: PLoS One. 2018 Sep 12;13(9):e0202870. doi: 10.1371/journal.pone.0202870 (PMC6135387; doi:10.1371/journal.pone.0202870)
Supplement: S1 Fig — Part A. Effect of rainforest patch size on vertebrate seed predator abundance. Abundance calculated as the percent camera days that a taxon was recorded (sampling rate) at a given site. Circles represent fragments, squares represent continuous forest (N = 6 in each). Continuous rainforest sites are contiguous with extensive drier eucalypt forest, while fragments are surrounded by pasture. Part B. Effect of rainforest patch size on vertebrate seed predator feeding behaviour. Feeding behaviour calculated as the percent camera days that a taxon was recorded physically interfering with seeds (destructive interest), calculated from the total number of days in which it was recorded at a given site. Circles represent fragments, squares represent continuous forest (N = 6 in each). Continuous rainforest sites are contiguous with extensive drier eucalypt forest, while fragments are surrounded by pasture. (DOCX) [file pone.0202870.s004.docx]

**S1 Fig. Part A. Effect of rainforest patch size on vertebrate seed predator abundance.**

**S1 Fig. Part B. Effect of rainforest patch size on vertebrate seed predator feeding behaviour.**
